# Fructose and glucose from sugary drinks enhance colorectal cancer metastasis via SORD

**DOI:** 10.1038/s42255-025-01368-w

**Published:** 2025-09-19

**Authors:** Tianshi Feng, Qin Luo, Yanlin Liu, Zeyu Jin, David Skwarchuk, Rumi Lee, Miso Nam, John M. Asara, Daya R. Adye, Philip L. Lorenzi, Lin Tan, Guangsheng Pei, Zhongming Zhao, Neda Zarrin-Khameh, Adriana Paulucci-Holthauzen, Brian W. Simons, Ju-Seog Lee, Scott Kopetz, Jihye Yun

**Affiliations:** 1 Department of Genetics, The University of Texas MD Anderson Cancer Center, Houston, TX USA; 2 Department of Molecular and Human Genetics, Baylor College of Medicine, Houston, TX USA; 3 Department of Cancer Biology, The University of Texas MD Anderson Cancer Center, Houston, TX USA; 4 Division of Signal Transduction, Beth Israel Deaconess Medical Center and Department of Medicine, Harvard Medical School, Boston, MA USA; 5 Metabolomics Core Facility, Department of Bioinformatics and Computational Biology, The University of Texas MD Anderson Cancer Center, Houston, TX USA; 6 Center for Precision Health, McWilliams School of Biomedical Informatics, The University of Texas Health Science Center at Houston, Houston, TX USA; 7 Department of Pathology & Immunology, Baylor College of Medicine, Houston, TX USA; 8 Center for Comparative Medicine, Baylor College of Medicine, Houston, TX USA; 9 Department of Systems Biology, The University of Texas MD Anderson Cancer Center, Houston, TX USA; 10 Department of Gastrointestinal Medical Oncology, The University of Texas MD Anderson Cancer Center, Houston, TX USA

**Keywords:** Cancer metabolism, Colorectal cancer, Metabolism, Metabolomics

## Abstract

The consumption of sugar-sweetened beverages (SSBs), which contain high levels of fructose and glucose, has been causally and mechanistically linked to an increased risk of colorectal cancer (CRC). However, the effects of SSB consumption on advanced stages of disease progression, including metastasis, remain poorly understood. Here we show that exposure of CRC cells to a glucose and fructose formulation—reflecting the composition of both high-fructose corn syrup and sucrose found in SSBs—enhances cellular motility and metastatic potential compared to glucose alone. Given that CRC cells grow poorly in fructose alone, and cells in vivo are not physiologically exposed to fructose without glucose, we excluded the fructose-only condition from our studies unless needed as a control. Mechanistically, the combination of glucose and fructose elevates the NAD⁺/NADH ratio by activation of the reverse reaction of sorbitol dehydrogenase in the polyol pathway. This redox shift relieves NAD⁺ limitations and accelerates glycolytic activity, which in turn fuels activation of the mevalonate pathway, ultimately promoting CRC cell motility and metastasis. Our findings highlight the detrimental impact of SSBs on CRC progression and suggest potential dietary and therapeutic strategies to mitigate metastasis in patients with CRC.

## Abstract (web summary)

Feng, Luo and colleagues show that the combination of glucose and fructose, as found in sugar-sweetened beverages, promotes colorectal cancer metastasis through a mechanism involving sorbitol dehydrogenase (SORD).

## Main

Diet is widely recognized as one of the most significant environmental factors contributing to the development of CRC^1^. Among the dietary factors that have gained increasing attention is the consumption of SSBs. SSBs are any liquids sweetened with added sugars, including sucrose and high-fructose corn syrup, both of which contain glucose and fructose in an approximate 1:1 ratio^2^. Since the 1980s, SSB consumption has risen globally^3^. In the USA, over half of adults and nearly two-thirds of youth consume SSBs daily^4,5^. This surge parallels alarming increases in young-onset CRC incidence and mortality^6,7^.

Our prior research using precancerous Apc-mutant mouse models, which mimic the early stages of CRC (adenomas)^8^, showed that SSBs can directly promote tumorigenesis independent of obesity^9^. A large epidemiological study later confirmed our findings, showing that SSB intake doubled the risk of young-onset CRC^10^. However, whether SSB consumption affects the later stages of CRC (carcinomas) remains unclear. Although some cohort studies suggest links between SSB intake and CRC recurrence or mortality, causal and mechanistic insights are lacking^11,12^. Addressing this question is essential, given that many patients with CRC are diagnosed at advanced stages, and some continue to consume SSBs even after diagnosis. Moreover, oncologists and nutritionists often recommend energy drinks and concentrated juices, which are essentially SSBs owing to their high glucose and fructose content. Therefore, we investigated whether SSB consumption promotes progression and metastasis of advanced CRC and explored the underlying mechanisms.

### Glucose and fructose together enhance CRC motility and metastasis

**Fig. 1:**
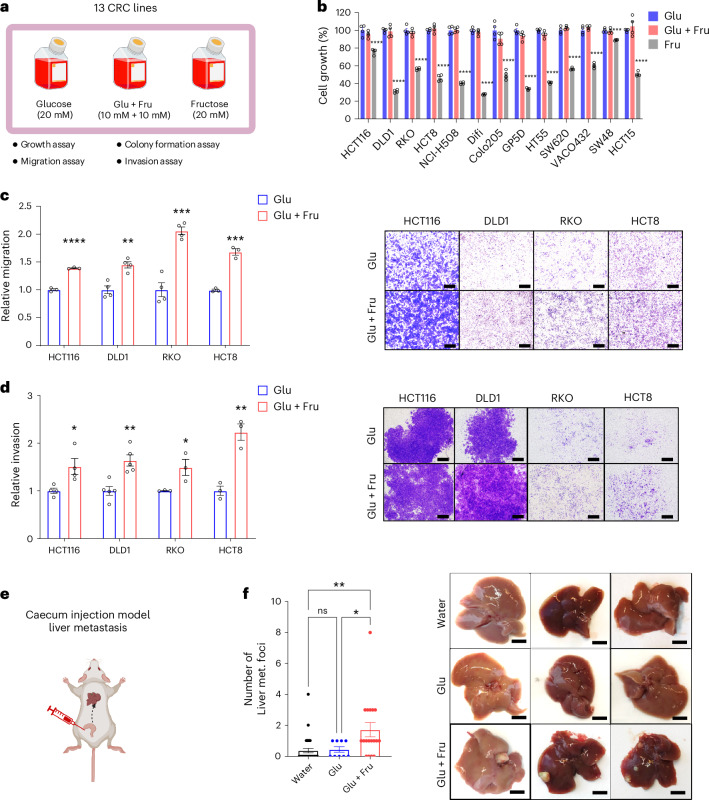
Glucose and fructose together enhance the migration, invasion and metastatic potential of CRC cells in vitro and in vivo*.* **a**, Schematic of cell culture medium used for multiple assays exploring the specific effects of glucose + fructose treatment across 13 CRC cell lines. **b**, Cell growth assay conducted with 20 mM glucose (Glu), 10 mM glucose + 10 mM fructose (Glu + Fru) or 20 mM fructose (Fru) in medium containing 10% dialysed FBS for 72 h. Values are normalized to the Glu condition (*n* = 4). **c**, Transwell migration assay of CRC cells conducted under Glu and Glu + Fru conditions. Migration was quantified by stained area, normalized to the Glu condition (HCT116, *n* = 3; DLD1, RKO, HCT8, *n* = 4). Representative images of migrated cells are shown in the right panel; scale bars, 500 μm. **d**, Transwell invasion assay of CRC cells conducted under Glu and Glu + Fru conditions. Invasion was quantified by stained area, normalized to the Glu condition (HCT116, *n* = 4; DLD1, *n* = 5; RKO, *n* = 3; HCT8, *n* = 3). Representative images of invading cells are shown in the right panel; scale bars, 500 μm. **e**, Schematic of the caecum injection model. **f**, Number of macroscopic liver tumour foci in athymic nude mice that received caecum injection of HCT116 cells and were treated with water (*n* = 32), 25% (w/v) Glu (*n* = 9) or 25% (w/v) Glu + Fru (45:55 ratio; *n* = 18) for 5 weeks; met., metastatic. Representative images from each group are shown in the right panel; scale bars, 0.5 cm. Data are presented as means and represent biological replicates; error bars, s.e.m. Statistical significance was determined by two-way ANOVA with Holm-Šídák post hoc test (**b**); one-way ANOVA with Dunnett’s post hoc test (**f**) or a two-tailed unpaired Student’s *t*-test (**c** and **d**). ****P < 0.0001; ***P < 0.001; **P < 0.01; *P < 0.05; n.s., not significant. Illustrations in **a** and **e** were created in BioRender.com. Source data

We previously showed that administering a glucose–fructose mixture in liquid form to Apc-mutant mice, mimicking SSB intake, exposes intestinal tumour cells to both sugars at millimolar (mM) concentrations, enabling efficient uptake^9^. In this study, we refer to the glucose–fructose mixture treatment as the ‘Glu + Fru’ condition, reflecting the physiological impact of high-fructose corn syrup or sucrose, both of which contain glucose and fructose. To examine the specific effects of this combination on advanced CRC, we used 13 CRC cell lines (carcinomas) with various mutations and conducted a cell growth assay under three equimolar sugar conditions (Fig. 1a): glucose only (20 mM), Glu + Fru (10 mM glucose + 10 mM fructose) and fructose only (20 mM). We found that Glu + Fru did not significantly alter cell growth compared to glucose alone (Fig. 1b). Notably, all cell lines showed poor growth in the fructose-only condition (Fig. 1b), which does not reflect physiological conditions, as cells are never exposed to fructose without glucose. Therefore, we excluded the fructose-only condition from further studies unless needed as a control.

**Extended Data Fig. 1:**
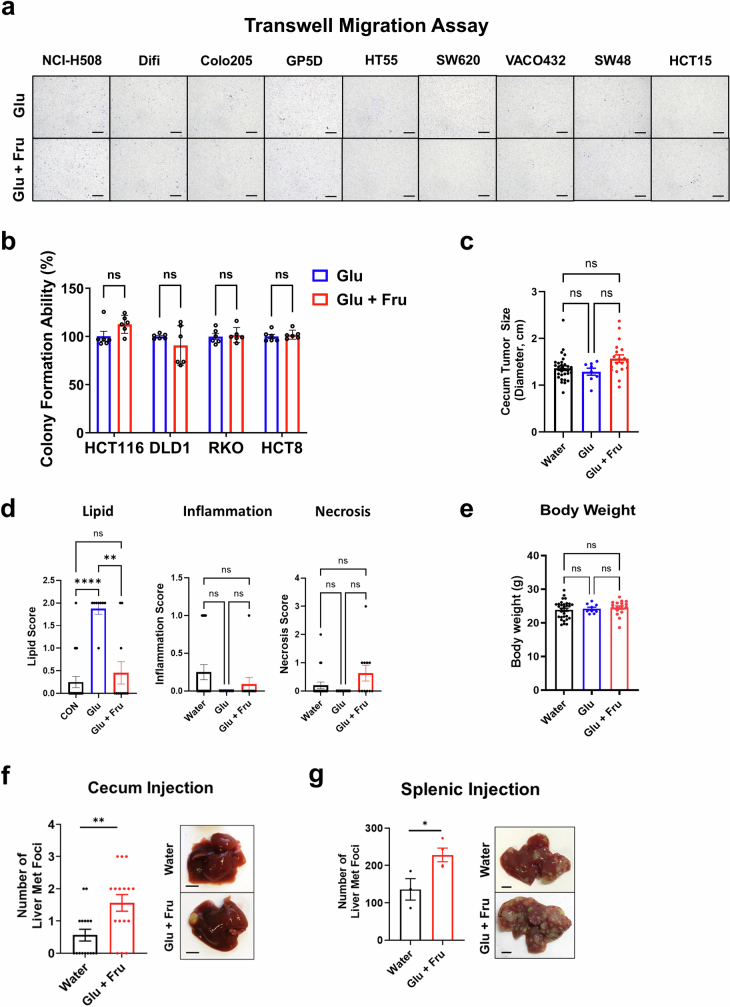
Glucose and fructose together enhance the migration, invasion, and metastatic potential of colorectal cancer cells in vitro and in vivo. **a**, Transwell images of 9 out of 13 CRC cell lines that did not migrate through the transwell under either 20 mM glucose (Glu) or 10 mM glucose + 10 mM fructose (Glu + Fru) with 10% dialyzed FBS for 48 h. The experiments were repeated twice, and representative images are shown. **b**, Colony formation assessed under 20 mM glucose (Glu) or 10 mM glucose + 10 mM fructose (Glu + Fru) (n = 6). **c**-**e**, Primary cecum tumor size (**c**), liver histological analysis (H&E) (**d**) and body weight (**e**) of athymic nude mice injected in the cecum with HCT116 cells and treated with water (Water; n = 31), 25% (w/v) glucose (Glu; n = 8), or 25% (w/v) glucose + fructose (45:55) (Glu + Fru; n = 19) in their drinking water for 5 weeks. For liver histology (**d**), n = 20 (Water), n = 8 (Glu), and n = 11 (Glu + Fru). **f**, The number of macroscopic liver tumor foci was quantified in mice that received either water or Glu + Fru following cecum injection of HCT116 cells. Mice were administered 400 μl water (Water) or 400 μl of 25% (w/v) Glu + Fru (45:55) daily via oral gavage for 5 weeks prior to liver metastasis assessment (n = 16 per group). Representative liver images are shown on the right; scale bar, 5 mm. **g**, The number of liver tumor foci was quantified in NSG mice that received intrasplenic injection of HCT116 cells and were treated with either water (Water; n = 3) or 25% (w/v) Glu + Fru (45:55) (Glu + Fru; n = 4) in their drinking water for 4 weeks. Representative liver images are shown on the right; scale bar, 5 mm. All data represent mean ± s.e.m. and are derived from biological replicates. Statistical analysis was performed using one-way ANOVA with Dunnett’s post hoc test (**c**, **e**); two-tailed unpaired Student’s t-test (**b**, **f**, **g**) or Kruskal-Wallis test (**d**). **P < 0.01; *P < 0.05; ns, not significant. Source data

Next, we conducted a transwell migration assay using 13 CRC cell lines. Nine cell lines did not migrate under either glucose-only or Glu + Fru conditions (Extended Data Fig. 1a), indicating that they lack intrinsic migratory ability. By contrast, the four lines that did migrate—HCT116, DLD1, RKO and HCT8—showed enhanced migration under Glu + Fru conditions compared to the glucose-only condition (Fig. 1c). Consistent with migration results, Glu + Fru also increased invasion compared to glucose, without affecting colony-forming ability (Fig. 1d and Extended Data Fig. 1b). These findings suggest that migratory CRC lines exposed to glucose and fructose uniquely acquire enhanced migration and invasion, key early steps of metastasis.

To investigate whether glucose and fructose together promote CRC metastasis in vivo, we used an orthotopic caecum injection model to assess local invasion and spontaneous liver metastasis (Fig. 1e). HCT116 cells were injected into the caecal submucosa of athymic nude mice, which then received water, glucose (25%) or Glu + Fru (25%) in drinking water for 5 weeks. Glu + Fru significantly increased liver metastasis, measured by liver metastatic foci, compared to water or glucose (Fig. 1f), without affecting primary tumour size (Extended Data Fig. 1c). Liver histology and body weight were unchanged between Glu + Fru and water groups (Extended Data Fig. 1d,e), suggesting that Glu + Fru alters neither the liver microenvironment nor body weight under these conditions. Moreover, daily oral gavage of moderate Glu + Fru significantly increased liver metastasis compared to the water group, with metastatic potential comparable to that of the Glu + Fru drinking water group (Extended Data Fig. 1f). Additionally, we confirmed these findings using an intrasplenic injection model, which showed that Glu + Fru enhanced liver metastasis independent of local invasion (Extended Data Fig. 1g). Collectively, these results suggest glucose and fructose together promote CRC metastasis more than glucose alone.

### Glucose and fructose together drive the SORD reverse reaction

**Fig. 2:**
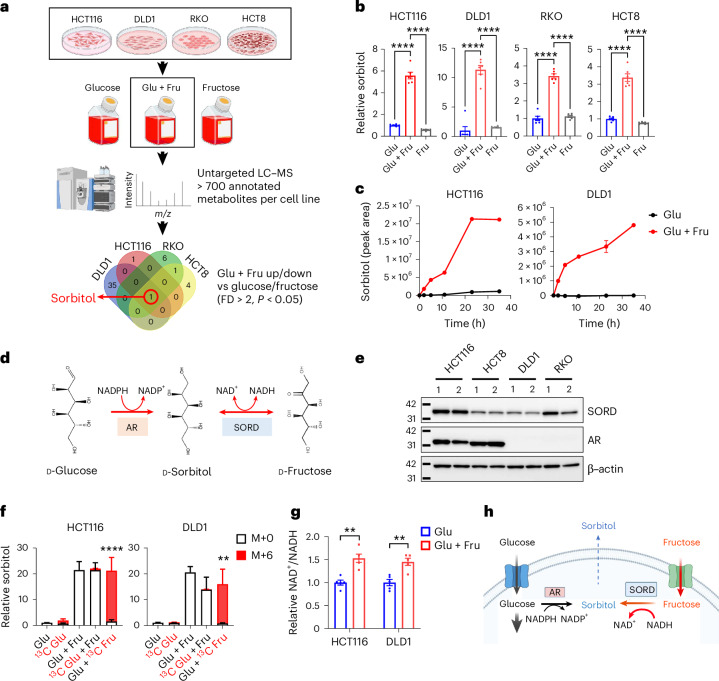
Glucose and fructose together activate the SORD reverse reaction, elevating sorbitol and NAD^+^/NADH ratios. **a**, Schematic of the metabolomics workflow in four CRC cell lines cultured for 48 h under three different conditions: 20 mM glucose, 10 mM glucose + 10 mM fructose and 20 mM fructose. The Venn diagram highlights metabolites commonly altered under Glu + Fru conditions (fold difference (FD) of >2; *P* < 0.05; *n* = 6). **b**, Relative intracellular sorbitol levels measured by LC–MS, normalized to the Glu group (*n* = 6). **c**, Intracellular sorbitol production after incubation in 20 mM glucose (*n* = 3) or 10 mM glucose + 10 mM fructose (*n* = 3). Cells were seeded in DMEM with 10% FBS, pre-treated with Glu or Glu + Fru medium containing 10% dialysed FBS for 1 h and then collected at different incubation time points. Sorbitol levels were measured as peak areas by LC–MS, with the average 0 h value set to zero. **d**, Schematic of the polyol (sorbitol) metabolic pathway. **e**, Immunoblotting of SORD and AR enzymes in four CRC cell lines. Cells were incubated with 20 mM glucose (labelled as 1) or 10 mM glucose + 10 mM fructose (labelled as 2) in DMEM with 10% dialysed FBS for 48 h. **f**, Sorbitol levels measured after 14 h of incubation with 20 mM glucose, 20 mM U-[^13^C]-glucose (^13^C Glu), 10 mM glucose + 10 mM fructose, 10 mM U-[^13^C]-glucose + 10 mM fructose (^13^C Glu + Fru) or 10 mM U-[^13^C]-fructose + 10 mM glucose (Glu + ^13^C Fru) in DMEM with 10% dialysed FBS, using LC–MS. M+6 denotes sorbitol fully labelled with six ^13^C carbons. Data were normalized to the Glu group. Statistical comparison of M+6 sorbitol levels between the ^13^C Glu + Fru and Glu + ^13^C Fru groups is shown (*n* = 3). **g**, Relative intracellular NAD⁺/NADH ratio in CRC cells assessed by LC–MS after 48 h incubation under Glu or Glu + Fru conditions, normalized to the Glu group (*n* = 5). **h**, Schematic showing how glucose and fructose together activate the SORD reverse reaction: glucose fuels glycolysis, generating NADH, which, together with fructose, enables SORD to convert fructose into sorbitol while oxidizing NADH to NAD⁺. Data are presented as means and represent biological replicates; error bars, s.e.m. Statistical significance was determined by one-way ANOVA with Dunnett’s post-test (**b**); two-way ANOVA with Holm-Šídák post hoc test (**f**); or two-tailed unpaired Student’s *t*-test (**g**). ****P < 0.0001; **P < 0.01. Illustrations in **a** and **h** were created in BioRender.com. Source data

**Extended Data Fig. 2:**
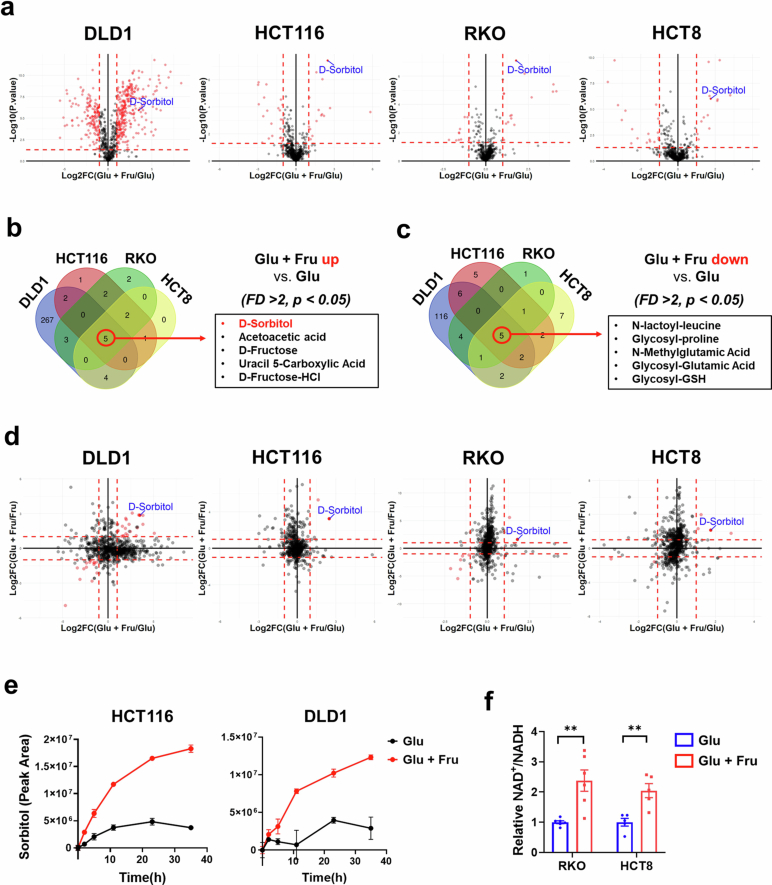
Sorbitol is significantly and consistently increased under glucose + fructose conditions compared to glucose or fructose alone across all four CRC cell lines. **a**, Volcano plots comparing metabolomic profiles from DLD1, HCT116, RKO, and HCT8 cells treated with 20 mM glucose (Glu) or 10 mM glucose + 10 mM fructose (Glu + Fru) in 10% dialyzed FBS for 48 h. Red dashed lines indicate –log₁₀(P value) = 1.3, log₂FC(Glu + Fru / Glu) = 1, and log₂FC(Glu + Fru / Glu) = –1. **b**, Venn diagram of commonly increased metabolites in Glu + Fru compared to Glu across all four CRC cell lines (fold change > 2; P < 0.05). **c**, Venn diagram of commonly decreased metabolites in Glu + Fru compared to Glu across all four CRC cell lines (fold change > 2; P < 0.05). **d**, Scatter plot showing metabolite changes under Glu + Fru versus glucose (x-axis) and Glu + Fru versus fructose (y-axis) in four CRC cell lines. Cutoff values: fold change > 2 and P < 0.05. Red dashed lines indicate log₂FC(Glu + Fru / Glu) = 1, log₂FC(Glu + Fru / Glu) = –1, log₂FC(Glu + Fru / Fru) = 1, and log₂FC(Glu + Fru / Fru) = –1. D-sorbitol is the only metabolite that meets the criteria for consistent enrichment across all four cell lines. **e**, Medium sorbitol levels following incubation in 20 mM glucose (Glu; n = 3) or 10 mM glucose + 10 mM fructose (Glu + Fru; n = 3). Cells were seeded in DMEM with 10% FBS, pre-treated with Glu or Glu + Fru medium containing 10% dialyzed FBS for 1 h, and collected at various timepoints. Sorbitol levels were measured as peak areas by LC/MS, with the average 0 h value set to zero. **f**, Relative intracellular NAD⁺/NADH ratio in CRC cells assessed by LC/MS after 48-hour incubation under Glu or Glu + Fru conditions, normalized to the Glu group. (RKO, n = 6; HCT8, n = 5). All data represent mean ± s.e.m. and are derived from biological replicates. Statistical analysis was performed using two-tailed unpaired Student’s t-test (**a** - **d** and **f**). **P < 0.01. Source data

To elucidate how the glucose–fructose combination promotes CRC migration and metastasis, we conducted global liquid chromatography–mass spectrometry (LC–MS) metabolomics on four CRC cell lines under glucose-only, Glu + Fru and fructose-only conditions (Fig. 2a). We aimed to identify metabolites consistently and uniquely altered by Glu + Fru across all four cell lines compared to glucose or fructose alone. The metabolomics platform identified over 700 annotated metabolites per cell line. Surprisingly, sorbitol was the only metabolite consistently and significantly increased more than twofold across all four CRC lines under Glu + Fru conditions, exhibiting threefold to 11-fold increases compared to glucose-only and fructose-only conditions (Fig. 2b and Extended Data Fig. 2a–d). We further confirmed that sorbitol was endogenously produced and then secreted over time following Glu + Fru treatment (Fig. 2c and Extended Data Fig. 2e).

Sorbitol is a sugar alcohol produced through the polyol pathway, a two-step process that converts glucose into fructose^13^ (Fig. 2d). In the first, irreversible step, glucose is reduced to sorbitol by aldose reductase (AR) using NADPH as a cofactor. The second, reversible step oxidizes sorbitol to fructose by sorbitol dehydrogenase (SORD), reducing NAD⁺ to NADH (Fig. 2d). Thus, elevated sorbitol under Glu + Fru could arise from glucose, fructose or both by glucose reduction by AR (NADPH-dependent) or fructose reduction by SORD in the reverse reaction (NADH-dependent). Notably, SORD was expressed in all four CRC lines, whereas AR was detected in HCT116 and HCT8 but absent in DLD1 and RKO (Fig. 2e). To determine which sugar is converted to sorbitol and which enzyme catalyses its formation under Glu + Fru conditions, we conducted stable ^13^C-isotope LC–MS experiments in HCT116 and DLD1 using three labelling conditions: ^13^C_6_-glucose-only, ^13^C_6_-glucose + unlabelled fructose and ^13^C_6_-fructose + unlabelled glucose. As expected, total sorbitol (labelled + unlabelled) was significantly higher under Glu + Fru than glucose-only (Fig. 2f), corroborating our unlabelled metabolomics (Fig. 2b). Remarkably, in cells incubated with ^13^C-fructose + unlabelled glucose, over 97% of sorbitol was fully ^13^C-labelled at all six carbons (M+6) (Fig. 2f). By contrast, with ^13^C-glucose + unlabelled fructose, sorbitol remained mostly unlabelled (Fig. 2f). No intermediate labelling (M+1, M+2, M+3, M+4 or M+5) was detected from either sugar, confirming direct conversion to sorbitol. These results indicate that elevated sorbitol under Glu + Fru is primarily derived from fructose through SORD, not glucose through AR, even in cell lines with high AR expression, such as HCT116.

Given that the SORD reverse reaction was activated under Glu + Fru versus glucose-only conditions, we reasoned that the NAD⁺/NADH ratio would likewise increase with sorbitol levels. Indeed, the NAD⁺/NADH ratio was significantly higher in all four CRC lines under Glu + Fru than glucose (Fig. 2g and Extended Data Fig. 2f). The fructose-only condition did not increase sorbitol (Fig. 2b), probably because of inefficient glycolytic entry and thus inadequate NADH production for the SORD reverse reaction. By contrast, under Glu + Fru conditions, glucose efficiently enters glycolysis, generating NADH through glyceraldehyde-3-phosphate dehydrogenase (GAPDH), while fructose directly serves as a substrate for SORD, together activating the reverse reaction (Fig. 2h).

### SORD expression is elevated in human colorectal tumours

**Fig. 3:**
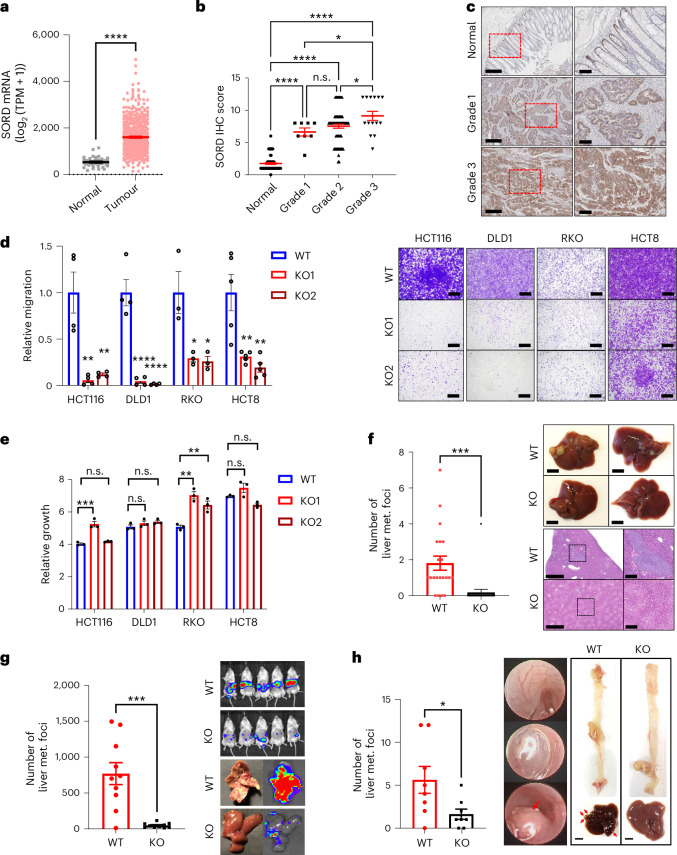
SORD mediates glucose–fructose-driven CRC motility and metastasis. **a**, SORD mRNA expression in normal tissues (*n* = 51) and colorectal tumours (*n* = 612) in The Cancer Genome Atlas Pan-Cancer dataset. **b**, SORD protein expression by IHC in normal tissues (*n* = 59) and in colorectal tumours of different grades (Grade 1, *n* = 8; Grade 2, *n* = 72; Grade 3, *n* = 15), assessed by staining intensity (0–4) and coverage. **c**, Representative IHC images of SORD in CRC samples. Red dashed rectangles in left panels represent zoomed-in images in right panels; scale bars, 400 μm (left) and 100 μm (right). **d**, Migration of WT and two independent SORD knockout clones (KO1 and KO2) under Glu + Fru conditions, assessed by transwell assay (HCT116, *n* = 4; DLD1, *n* = 4; RKO, *n* = 3; HCT8, *n* = 5). Values were normalized to WT. Right, representative images; scale bar, 500 μm. **e**, Growth of WT, KO1 and KO2 cells (*n* = 3) under Glu + Fru conditions, assessed by CellTiter-Glo. Cell numbers at 96 h were normalized to values at 24 h. **f**, Macroscopic liver metastases from caecum tumours generated with HCT116 SORD WT (*n* = 21) or KO (*n* = 23) cells in athymic nude mice provided with 25% (w/v) Glu + Fru (45:55) in drinking water for 5 weeks. Top right, representative liver images; scale bar, 5 mm. Bottom right, H&E staining. Black dashed squares in left panels represent zoomed-in images in right panels; scale bars, 1 mm (left), 200 μm (right). **g**, Liver metastases in NSG mice injected in the spleen with luciferase-labelled SORD WT or KO HCT116 cells (*n* = 10 per group), followed by 5 weeks of 25% (w/v) Glu + Fru (45:55) in drinking water. Right, bioluminescence imaging on day 17. Representative H&E staining of liver metastases is shown in Extended Data Fig. 4i. **h**, Macroscopic liver metastases from NSG mice receiving colonic injection of SORD WT or KO HCT116 cells (*n* = 8 per group), followed by 4 weeks of 25% (w/v) Glu + Fru (45:55) in drinking water. Middle, colonoscopic images before, during and 2 weeks after injection. Right, representative images of colon and liver; red arrows indicate metastases; scale bar, 5 mm. Data are presented as mean and represent biological replicates; error bars, s.e.m. Statistical analysis was performed using two-tailed unpaired Student’s *t*-test (**a** and **f**–**h**) or one-way ANOVA with Dunnett’s post hoc test (**b**, **d** and **e**). ****P < 0.0001; ***P < 0.001; *P < 0.01; *P* < 0.05*;* n.s., not significant. Source data

**Extended Data Fig. 3:**
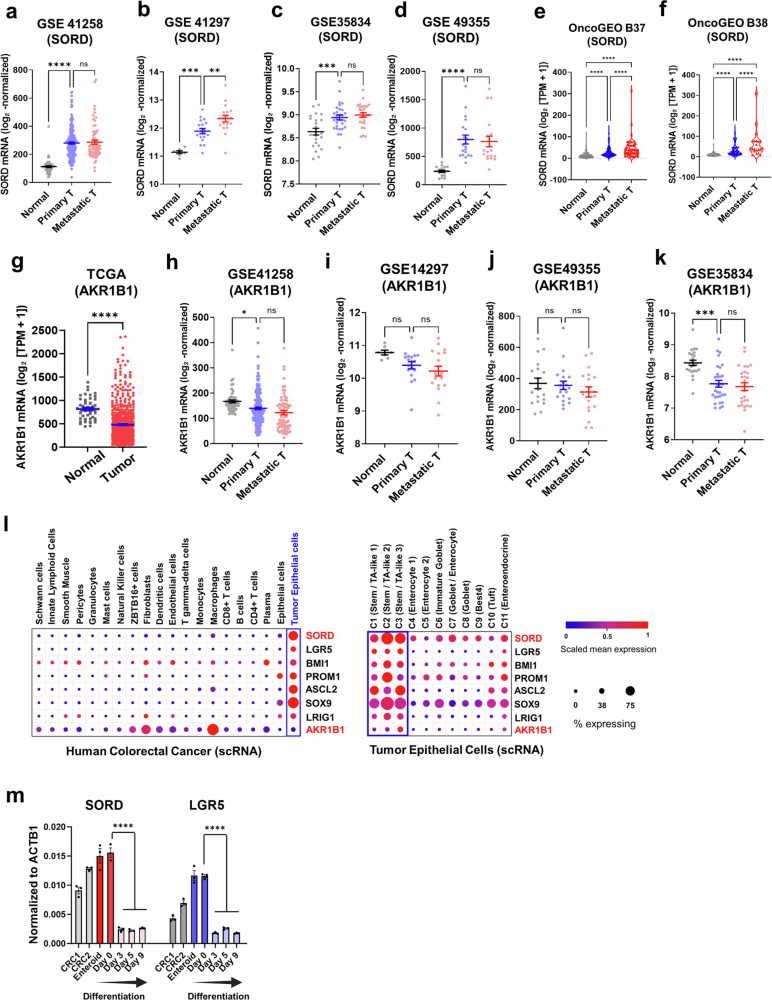
SORD expression, but not AKR1B1 expression, is elevated in human colorectal tumors. **a**, SORD mRNA expression in normal tissues (n = 54), primary colorectal tumors (n = 185), and metastatic colorectal tumors (n = 67) in GSE41258. **b**, SORD mRNA expression in normal tissues (n = 7), primary colorectal tumors (n = 18), and metastatic colorectal tumors (n = 18) in GSE14297. **c**, SORD mRNA expression in normal tissues (n = 23), primary colorectal tumors (n = 30), and metastatic colorectal tumors (n = 27) in GSE35834 dataset. **d**, SORD mRNA expression in normal tissues (n = 18), primary colorectal tumors (n = 20), and metastatic colorectal tumors (n = 19) in GSE49355. **e**, SORD mRNA expression in normal tissues (n = 162), primary colorectal tumors (n = 233), and metastatic colorectal tumors (n = 55) in OncoGEO B37. **f**, SORD mRNA expression in normal tissues (n = 74), primary colorectal tumors (n = 137), and metastatic colorectal tumors (n = 21) in OncoGEO B38 dataset. **g**, AKR1B1 mRNA expression in normal tissue (n = 51) and colorectal tumor tissues (n = 612) in TCGA PANCAN dataset. One outlier (value > mean + 6σ) in the tumor group was excluded from the graph, which did not affect statistical significance. **h**, AKR1B1 mRNA expression in normal tissues (n = 54), primary colorectal tumors (n = 185), and metastatic colorectal tumors (n = 67) in GSE41258 dataset. **i**, AKR1B1 mRNA expression in normal tissues (n = 7), primary colorectal tumors (n = 18), and metastatic colorectal tumors (n = 18) in GSE14297 dataset. **j**, AKR1B1 mRNA expression in normal tissues (n = 18), primary colorectal tumors (n = 20), and metastatic colorectal tumors (n = 19) in GSE49355 dataset. **k**, AKR1B1 mRNA expression in normal tissues (n = 23), primary colorectal tumors (n = 30), and metastatic colorectal tumors (n = 27) in GSE35834 dataset. **l**, Dot plot of SORD, AKR1B1 and intestinal stem cell markers expression across all clusters (left) and tumor epithelial clusters (right) in the Human Colon Cancer Atlas (c295) single-cell dataset. Dot size indicates the percentage of cells expressing the gene; color intensity reflects average scaled expression. **m**, Relative expression of SORD and LGR5 in human CRC organoids, human enteroids, and during enteroid monolayer differentiation (Days 0, 3, 5, and 9) (n = 3). All data represent mean ± s.e.m. and are derived from biological replicates. Statistical analysis was performed using one-way ANOVA with Dunnett’s post hoc test (**a**-**f**, **h**-**k**, **m**) or two-tailed unpaired Student’s t-test (**g**). ****P < 0.0001; ***P < 0.001; *P < 0.05; ns, not significant. Source data

Supporting our findings, SORD mRNA and protein levels were significantly higher in human colorectal tumours than in adjacent normal epithelium (Fig. 3a–c and Extended Data Fig. 3a–f), consistent with previous proteomics studies^14,15^. In some cohorts, metastatic tumours exhibited higher SORD mRNA levels than primary tumours (Extended Data Fig. 3b,e,f). Higher-grade tumours also showed elevated SORD protein levels compared to lower-grade tumours (Fig. 3b,c). By contrast, AKR1B1 mRNA, encoding AR in the polyol pathway, was downregulated or unchanged in colorectal tumours versus normal tissue (Extended Data Fig. 3g–k), suggesting that SORD—not AR—may have a more prominent role in CRC development.

The heterogeneity of CRC tissue—including immune, stromal and epithelial cells—complicates transcriptional analysis from bulk tumour samples. To address this limitation, we analysed single-cell RNA sequencing (scRNA-seq) profiles from 62 CRC and 36 adjacent normal tissue samples^16^. SORD expression—unlike AKR1B1—was predominantly elevated in tumour epithelial cells across 20 clusters, with minimal expression in immune and stromal cells, confirming tumour-specific upregulation (Extended Data Fig. 3l). Importantly, SORD was highly expressed in stem-cell/TA-like epithelial subtypes (Extended Data Fig. 3l). Its expression resembled that of known intestinal stem cell markers (LGR5, BMI1), suggesting a stem-cell-like role for SORD (Extended Data Fig. 3l). We further validated that SORD expression was high in undifferentiated, stem-like human colon enteroids but decreased upon differentiation, mirroring LGR5 downregulation (Extended Data Fig. 3m). Two patient-derived CRC organoids exhibited higher SORD expression than differentiated colonic epithelial cells, consistent with patient mRNA data (Extended Data Fig. 3m). Given the known link between stem-like tumour cells and increased aggressiveness^17^, these findings suggest a role for SORD in CRC migration and metastasis.

### SORD mediates glucose–fructose-driven CRC motility and metastasis

**Extended Data Fig. 4:**
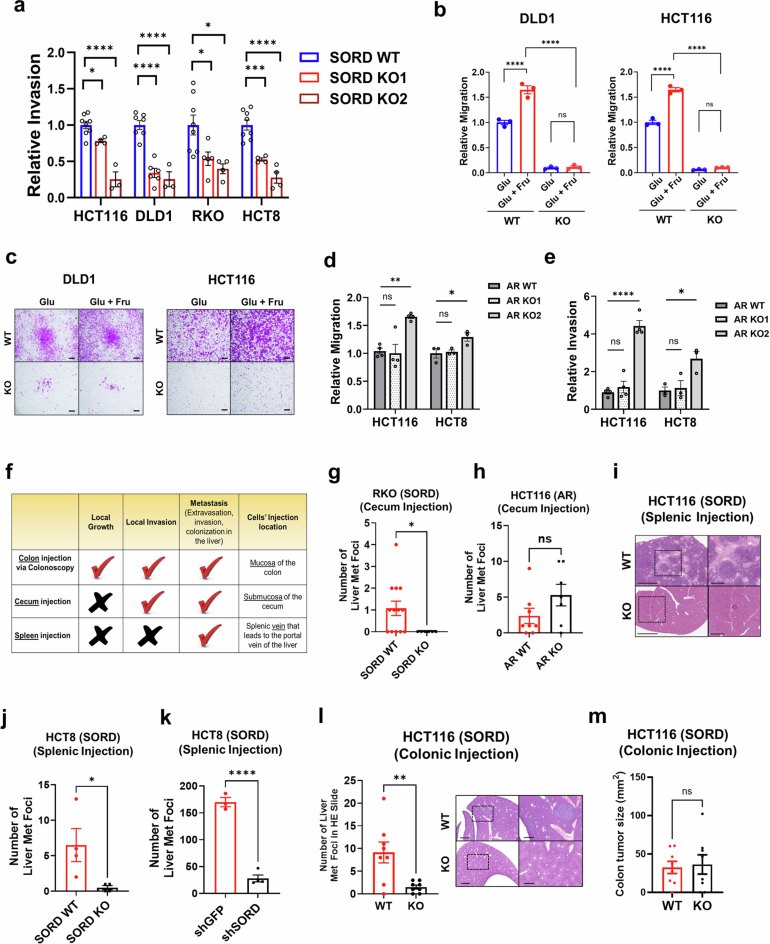
SORD, but not AR, mediates glucose–fructose-driven colorectal cancer (CRC) migration, invasion, and metastasis. **a**, Transwell invasion assay of isogenic SORD WT (Cas9-expressing control) and two knockout (KO) clones derived from four CRC cell lines under Glu + Fru conditions. Invasion was quantified based on stained area and normalized to WT (SORD WT: HCT116, RKO and HCT8: n = 8; DLD1, n = 7. SORD KO1: HCT116 and HCT8: n = 4; DLD1: n = 6; RKO: n = 5. SORD KO2: HCT116 and DLD1: n = 3; HCT8 and RKO: n = 5). **b**, **c**, Transwell migration assay (**b**) and representative images (**c**) of wild-type (WT; n = 3) and SORD KO (n = 3) cells under Glu or Glu + Fru conditions. Migration was quantified based on stained area and normalized to WT-Glu. Scale bar, 500 μm. **d**, Transwell migration assay of isogenic aldose reductase (AR) WT (Cas9-expressing control) and two AR KO clones derived from HCT116 (n = 4) and HCT8 (n = 3) under Glu + Fru condition. Values were normalized to WT. **e**, Transwell invasion assay of isogenic AR WT and two KO clones derived from HCT116 (n = 4) and HCT8 (n = 3) under Glu + Fru condition. Values were normalized to WT. **f**, Schematic overview of tumor progression features evaluated using each xenograft model. Checkmarks indicate which biological processes—local growth, invasion, and metastasis—can be assessed based on injection site and model design. **g**, Number of macroscopic liver tumor foci from cecum tumors established with RKO-derived SORD WT (n = 13) or KO (n = 6) cells. **h**, Number of macroscopic liver tumor foci from cecum tumors established with HCT116-derived AR WT (n = 8) or KO (n = 7) cells. **i**, Representative H&E staining from Fig. 3g showing liver metastases in NOD scid gamma (NSG) mice injected in the spleen with luciferase-labeled SORD WT or KO HCT116 cells. Scale bars, 3 mm (left), 1 mm (right). **j**, Number of macroscopic liver tumor foci in mice injected with SORD WT and KO HCT8 cells in the splenic injection model (n = 4 per group). **k**, Number of macroscopic liver tumor foci in GFP-shRNA (n = 3) and SORD-shRNA2 (n = 4) groups derived from HCT8 in a splenic injection model. **l**, Microscopic liver metastases from NSG mice receiving colonic injection of SORD WT or KO HCT116 cells (n = 8 per group), followed by 4 weeks of 25% (w/v) Glu + Fru (45:55) in drinking water. Right: representative H&E staining; scale bars, 1 mm (left), 400 μm (right). **m**, Colon tumor size in NSG mice injected with SORD WT (n = 8) or KO (n = 8) HCT116 cells into the distal colon mucosa under colonoscopic guidance. All data represent mean ± s.e.m. and are derived from biological replicates. All mice receiving in vivo injections were treated with 25% (w/v) glucose + fructose (45:55) in drinking water for 5 weeks unless otherwise specified. Statistical analysis was performed using one-way ANOVA with Dunnett’s post hoc test (**a**, **b**, **d**, **e**) or two-tailed unpaired Student’s t-test (**g**–**m**). ****P < 0.0001; ***P < 0.001; **P < 0.01; *P < 0.05; ns, not significant. Source data

Given the SORD reverse reaction activation under Glu + Fru and elevated SORD expression in human CRC, we reasoned that SORD is essential for the motility and metastasis promoted by glucose and fructose. To test this idea, we generated two independent CRISPR–Cas9 SORD knockout (KO) clones in four CRC cell lines. SORD KO cells showed reduced migration and invasion compared to parental and Cas9 control cells (SORD wild-type (WT)) (Fig. 3d and Extended Data Fig. 4a), while maintaining comparable growth rates (Fig. 3e). Notably, SORD KO cells reduced migration under both glucose-only and Glu + Fru (Extended Data Fig. 4b,c), suggesting an intrinsic role in motility independent of sugar. Although the mechanism remains unclear, SORD KO cells completely abolished the Glu + Fru-induced increase in migration, underscoring SORD’s essential role in motility enhanced by glucose and fructose. By contrast, AR KO cells in HCT116 and HCT8, which exhibit high AR expression, did not impair migration or invasion (Extended Data Fig. 4d,e).

To validate our findings in vivo, we used three complementary xenograft metastasis mouse models (Extended Data Fig. 4f). First, in the caecum injection model, Glu + Fru failed to increase liver metastasis in mice injected with SORD KO cells compared to those with SORD WT cells (Fig. 3f and Extended Data Fig. 4g). By contrast, AR KO cells did not abolish Glu + Fru-induced liver metastasis compared to AR WT cells (Extended Data Fig. 4h). Second, in the intrasplenic model, livers from mice with SORD WT cells had numerous metastases, whereas SORD KO or knockdown cells led to fewer foci and preserved histology (Fig. 3g and Extended Data Fig. 4i–k). Third, we used a colonic injection model, wherein cancer cells are injected into the mucosa of the colon using a specialized needle under colonoscopy guidance^18^. This model uniquely replicates all stages of the growth–invasion–metastasis cascade to the liver. Mice injected with SORD WT cells had more liver tumours than SORD KO cells, while primary colon tumour size was comparable (Fig. 3h and Extended Data Fig. 4l,m), indicating that SORD primarily affects metastasis, not primary tumour growth. Taken together, these orthogonal mouse experiments provide strong evidence that SORD is necessary for motility and metastasis augmented by glucose and fructose.

### Elevated NAD^+^/NADH ratio caused by SORD promotes CRC motility and metastasis

**Fig. 4:**
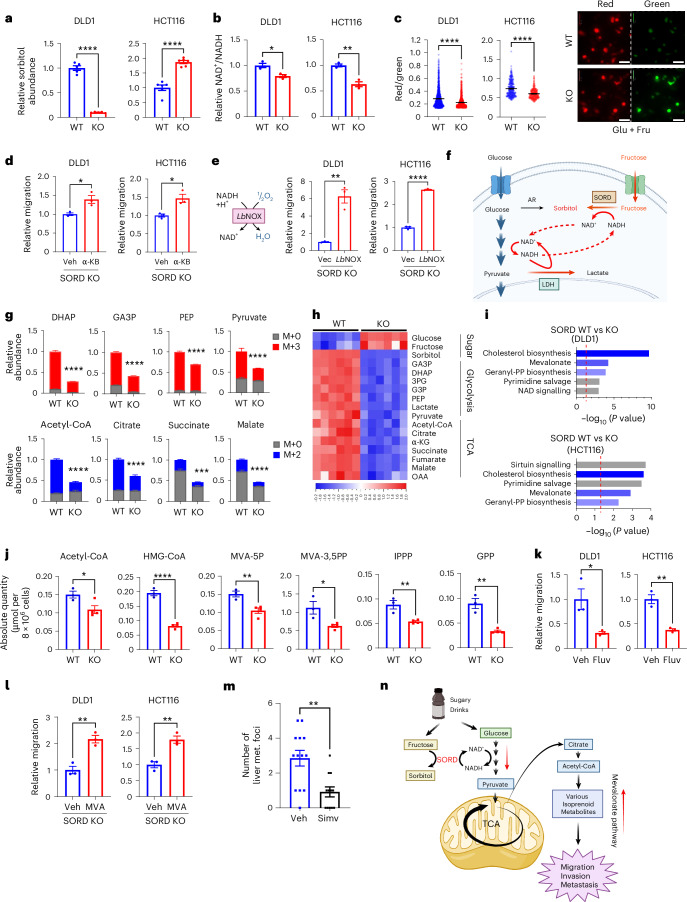
Elevated NAD^+^/NADH ratio caused by SORD promotes CRC motility and metastasis. **a**, Sorbitol levels in CRC cells after 48 h of incubation under Glu + Fru (10 mM each) conditions, measured by LC–MS (DLD1, *n* = 5; HCT116, *n* = 6). **b**, Relative NAD⁺/NADH ratio in CRC cells after 48 h of incubation under Glu + Fru conditions, measured by LC–MS (*n* = 3). **c**, Quantification of cytosolic Peredox NADH biosensor signals in WT and SORD KO CRC cells under Glu + Fru conditions after 24 h. The red/green fluorescence ratio reflects the NAD⁺/NADH ratio (DLD1 WT, *n* = 1205; DLD1 KO, *n* = 1147; HCT116 WT, *n* = 335; HCT116 KO, *n* = 311). Right, representative images; scale bars, 50 μm. **d**, Migration of SORD KO cells treated with vehicle (Veh) or α-ketobutyrate (α-KB) (DLD1, 1 mM; HCT116, 0.1 mM), assessed by transwell assay (*n* = 3). Values were normalized to the Veh group. **e**, Migration of SORD KO cells expressing empty vector (Vec) or *Lb*NOX, assessed by transwell assay (*n* = 3). Values were normalized to the Vec group. Left: schematic of the chemical reaction catalysed by *Lb*NOX. **f**, Schematic illustrating how SORD may enhance glycolysis under Glu + Fru conditions in CRC cells. The combination of glucose and fructose promotes regeneration of cytosolic NAD⁺, which is required to sustain high rates of aerobic glycolysis. **g**, LC–MS quantification of key glycolytic and TCA cycle metabolites in SORD WT and KO DLD1 cells after exposure to 10 mM U-[^13^C]-glucose and 10 mM fructose in 10% dialysed FBS. Sampling times, 10 min for DHAP, GA3P, PEP and pyruvate; 3 h for acetyl-CoA, citrate and succinate; 12 h for malate (*n* = 5). M+2 and M+3 indicate incorporation of two or three [^13^C] carbon atoms, respectively. **h**, Heatmap of relative metabolite levels in sugar metabolism, glycolysis and the TCA cycle in SORD WT and KO DLD1 cells under Glu + Fru conditions, measured by LC–MS (*n* = 6). Red indicates an increase; blue indicates a decrease. **i**, Metabolic pathways enriched in SORD WT versus KO cells (DLD1 and HCT116), based on RNA-seq data (*n* = 4). Cells were incubated under Glu + Fru conditions for 48 h. Pathway analysis was performed using Qiagen Ingenuity Pathway Analysis on genes with *P* < 0.05 and |log_2_(fold change)| > 0.5. Blue bars indicate mevalonate-related pathways. The *P* value and fold change were calculated using Qiagen Ingenuity Pathway Analysis with Fisher’s exact test. **j**, Absolute quantities of key mevalonate pathway metabolites in SORD WT and KO DLD1 cells under Glu + Fru conditions, measured by LC–MS (WT, *n* = 3; KO, *n* = 4). **k**, Migration of SORD WT cells assessed by transwell assay under Glu + Fru conditions with vehicle or fluvastatin (Fluv) (*n* = 3). Values were normalized to vehicle. DLD1, 1.5 μM; HCT116, 1 μM. **l**, Migration of SORD KO cells assessed by transwell assay with vehicle (or mevalonolactone (MVA; a cell-permeable form of mevalonate) (*n* = 3). Values were normalized to vehicle. DLD1, 5 mM; HCT116, 2 mM. **m**, Liver metastatic tumour foci counts in mice that received vehicle (*n* = 13) or simvastatin (Simv; *n* = 12) by daily oral gavage. Following caecal injection of HCT116 cells, athymic nude mice were treated with 100 μl day^−1^ of 10% DMSO (Veh) or 30 mg kg^−1^ day^−1^ simvastatin in 100 μl of 10% DMSO. All mice received 25% Glu + Fru (45:55) in drinking water for 5 weeks before liver metastasis assessment. **n**, Schematic showing how consumption of sugary drinks containing glucose and fructose may promote CRC migration and metastasis via SORD. All in vitro experiments were conducted under Glu + Fru (10 mM each) conditions unless otherwise specified. Data are presented as means and represent biological replicates; error bars, s.e.m. Statistical significance was determined by two-tailed unpaired Student’s *t*-test (**a**–**e** and **j**–**m**) or two-way ANOVA with Holm–Šídák post hoc test (**g**). ****P < 0.0001; ***P < 0.001; **P < 0.01; *P < 0.05. Illustration in **n** created with BioRender.com. LDH, lactate dehydrogenase; DHAP, dihydroxyacetone phosphate; GA3P, glyceraldehyde-3-phosphate; 3PG, 3-phosphoglycerate; G3P, glycerol-3-phosphate; PEP, phosphoenolpyruvate; OAA, oxaloacetate; HMG-CoA, 3-hydroxy-3-methylglutaryl-CoA; MVA-5P, mevalonate-5-phosphate; MVA-5PP, mevalonate-3,5-bisphosphate; IPPP, isopentenyl pyrophosphate; GPP, geranyl pyrophosphate. Source data

**Extended Data Fig. 5:**
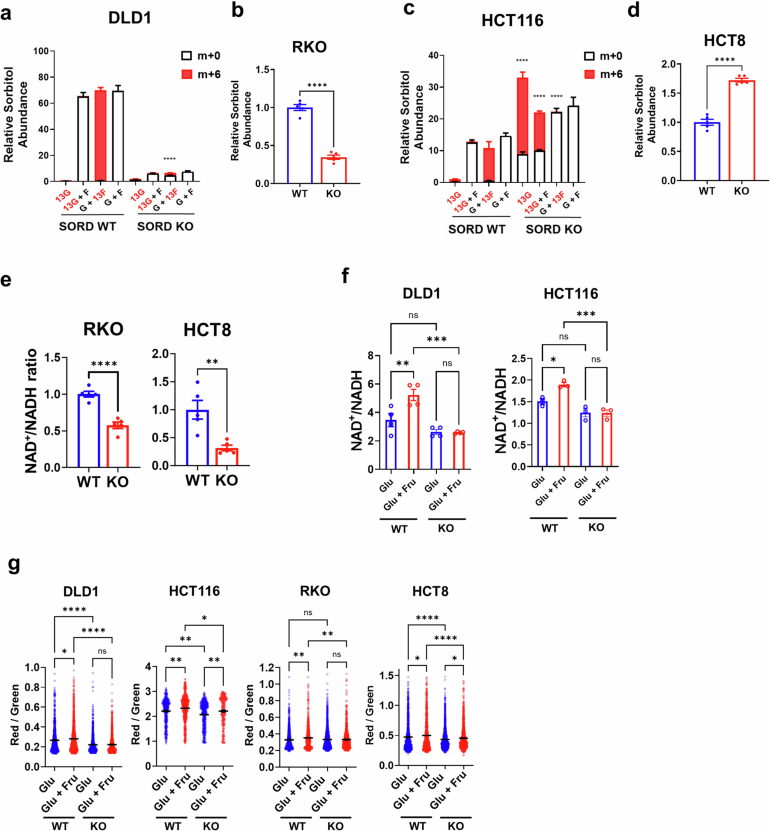
SORD mediates glucose–fructose-driven migration via the NAD⁺/NADH ratio, but not through sorbitol. **a**, Sorbitol levels in isogenic SORD WT and KO clones derived from DLD1 were measured after 14 h of incubation under the following conditions: 20 mM U-[13C]-glucose (13 G), 10 mM U-[13C]-glucose + 10 mM fructose (13 G + F), or 10 mM glucose + U-[13C]-fructose (G + 13 F), 10 mM glucose + 10 mM fructose (G + F), all in DMEM with 10% dialyzed FBS. Metabolite levels were quantified using LC/MS (n = 5). **b**, Sorbitol levels in SORD WT and KO clones derived from RKO after 48 h of incubation under Glu + Fru (10 mM each) conditions, measured by LC/MS (n = 5). **c**, Sorbitol levels in isogenic SORD WT and KO clones derived from HCT116 were measured (n = 3). The condition is the same as in (**a**). **d**, Measurement of relative sorbitol level in SORD WT and KO clones derived from HCT8. (n = 5). The condition is the same as in (**b**). **e**, Relative NAD⁺/NADH ratio in CRC cells after 48 h of incubation under Glu + Fru conditions, measured by LC/MS (n = 3). **f**, The NAD⁺/NADH ratio in SORD WT and KO cells cultured in 20 mM glucose (Glu) or 10 mM glucose + 10 mM fructose (Glu + Fru) was measured using an enzymatic assay kit (DLD1: n = 4; HCT116: n = 3). **g**, Quantification of the cytosolic Peredox NADH biosensor in WT and SORD KO cells from DLD1 (WT Glu, n = 960; WT Glu + Fru, n = 1205; KO Glu, n = 1264; KO Glu + Fru, n = 1147), HCT116 (WT Glu, n = 382; WT Glu + Fru, n = 509; KO Glu, n = 382; KO Glu + Fru, n = 247), RKO (WT Glu, n = 783; WT Glu + Fru, n = 646; KO Glu, n = 1506; KO Glu + Fru, n = 1161), and HCT8 (WT Glu, n = 994; WT Glu + Fru, n = 1013; KO Glu, n = 1489; KO Glu + Fru, n = 1554), after 24 h under Glu or Glu + Fru conditions. The Peredox biosensor (green) and mCherry (red) were quantified, and the red/green fluorescence ratio reflects the NAD⁺/NADH ratio. All data represent mean ± s.e.m. from biological replicates. Statistical significance was determined using two-way ANOVA with Holm–Sidak post hoc test (**a**, **c**), two-tailed unpaired Student’s t-test (**b**, **d**, **e**) or one-way ANOVA with Dunnett’s post hoc test (**f**, **g**). ****P < 0.0001; ***P < 0.001; **P < 0.01; *P < 0.05; ns, not significant. Source data

Given the activation of the SORD reverse reaction under Glu + Fru conditions and elevated SORD expression in human CRC, we reasoned that SORD is essential for motility and metastasis promoted by glucose and fructose. SORD KO cells from DLD1 and RKO, which lack AR expression, showed markedly lower sorbitol levels than WT cells under Glu + Fru conditions (Fig. 4a and Extended Data Fig. 5a,b). By contrast, SORD KO cells from HCT116 and HCT8, which express AR, showed higher sorbitol levels than WT cells under Glu + Fru conditions (Fig. 4a and Extended Data Fig. 5c,d). ^13^C-tracing showed that over 50% of sorbitol in SORD KO cells from HCT116 cell lines came from glucose (Extended Data Fig. 5c), suggesting AR activation upon SORD loss. Given the mismatch between sorbitol levels and migration ability in SORD WT and KO CRC cells, we excluded sorbitol as a common mediator of Glu + Fru-induced migration.

We next tested whether the elevated NAD^+^/NADH ratio from the SORD reverse reaction under Glu + Fru contributes to the increased CRC migration in SORD WT versus KO cells. Unlike sorbitol levels, the NAD^+^/NADH ratio—measured by LC–MS or enzymatic assays—was consistently lower in SORD KO than WT cells across all four CRC lines (Fig. 4b and Extended Data Fig. 5e,f). Although widely used, LC–MS or enzymatic assays require cell lysis and cannot distinguish between cytosolic and mitochondrial NAD^+^/NADH pools or between free and protein-bound forms. To address this limitation, we used Peredox, a fluorescent NAD^+^/NADH biosensor that detects the cytosolic redox state in live SORD isogenic cells^19^. Binding of NADH to the Peredox sensor increases green fluorescence without affecting the red fluorescence. Therefore, a lower free cytosolic NAD^+^/NADH ratio results in cells displaying increased green fluorescence. Indeed, SORD KO cells exhibited a decreased red-to-green ratio—indicative of a lower cytosolic NAD^+^/NADH ratio—relative to SORD WT cells across all four CRC cell lines (Fig. 4c and Extended Data Fig. 5g). Together, these results suggest that SORD may promote CRC motility by elevating the NAD^+^/NADH ratio.

**Extended Data Fig. 6:**
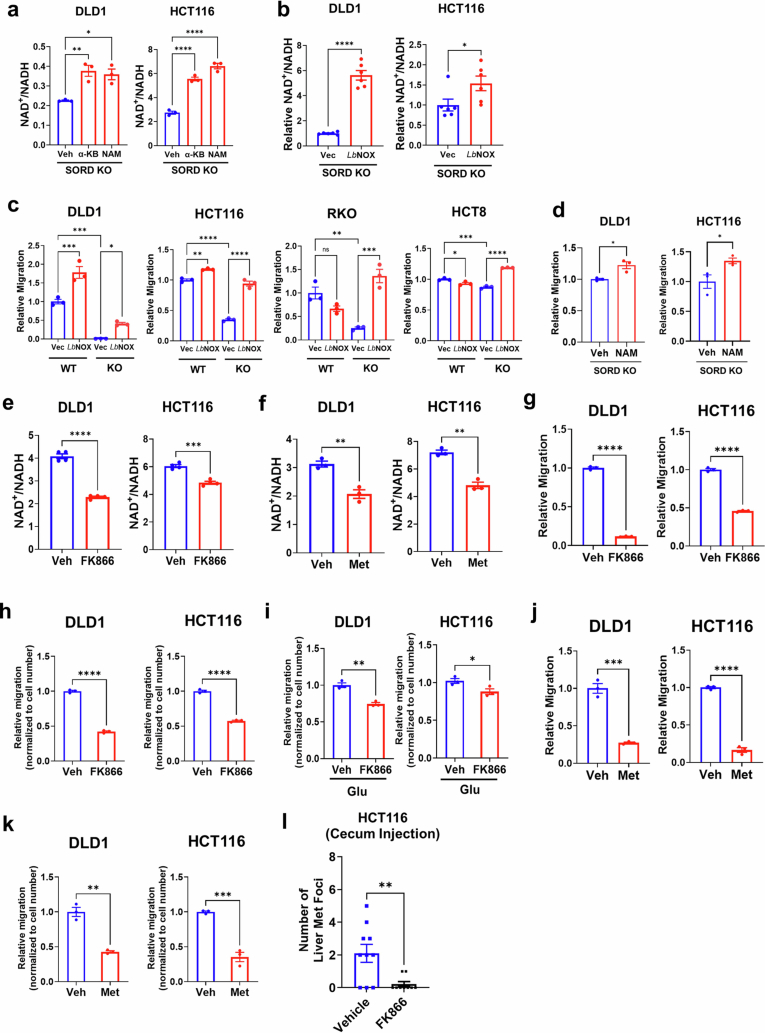
Elevated NAD+/NADH ratio caused by SORD promotes CRC motility. **a**, NAD⁺/NADH ratio in SORD KO cells measured by enzymatic bioluminescence assay after 24 h of treatment with vehicle, α-ketobutyrate (α-KB; DLD1: 1 mM; HCT116: 0.1 mM), or nicotinamide (NAM; DLD1: 2.5 mM; HCT116: 5 mM) (n = 3). **b**, NAD⁺/NADH ratio in SORD KO cells expressing either empty vector (Vec) or LbNOX, measured by LC/MS (n = 6). Values were normalized to Vec. **c**, Transwell migration assay of isogenic SORD WT and KO cells expressing either empty vector (Vec) or LbNOX (n = 3), derived from four CRC cell lines. Values were normalized to WT–Vec. **d**, Transwell migration assay of SORD KO cells treated with vehicle or NAM (DLD1: 2.5 mM; HCT116: 5 mM). Values were normalized to vehicle (n = 3). **e**, NAD⁺/NADH ratio in SORD KO cells measured by enzymatic assay after 24 h of treatment with vehicle or FK866 (DLD1: 10 nM; HCT116: 1 nM) (n = 3). **f**, NAD⁺/NADH ratio in SORD KO cells measured by enzymatic assay after 24 h of treatment with vehicle or metformin (1 mM) (n = 3). **g**, Migration of SORD WT cells treated with vehicle or FK866 (DLD1, 10 nM; HCT116, 1 nM), a NAMPT inhibitor, assessed by transwell assay (n = 3). **h**, Transwell migration assay of DLD1 (n = 3) and HCT116 (n = 3) cells treated with vehicle or FK866 (DLD1: 10 nM; HCT116: 1 nM). Drug concentrations were optimized for each cell line to minimize cytotoxicity while allowing assessment of migration effects. Cell numbers were measured in parallel under the same conditions to account for potential proliferation effects. Migration values were normalized to cell number using SYBR Green assay results and expressed as fold change relative to the vehicle group. **i**, Transwell migration assay of DLD1 (n = 3) and HCT116 (n = 3) cells under 20 mM glucose (Glu) with vehicle or FK866 (DLD1: 10 nM; HCT116: 1 nM). Migration values were normalized to cell number measurements and expressed as fold change relative to vehicle. **j**, Migration of SORD WT cells treated with vehicle or metformin (1 mM), assessed by transwell assay (n = 3). **k**, Migration values in (**j**) were normalized to cell number measurements and expressed as fold change relative to vehicle. **l**, Macroscopic liver tumor foci counts from cecal tumors established with HCT116 SORD WT cells treated with vehicle (n = 10) or FK866 (n = 9), administered by intraperitoneal injection (10 mg/kg body weight) every other day for 5 weeks under 25% (w/v) Glu + Fru (45:55) in drinking water. All in vitro experiments were conducted under Glu + Fru (10 mM each) conditions unless otherwise noted. All data represent mean ± s.e.m. from biological replicates. Statistical analysis was performed using one-way ANOVA with Dunnett’s post hoc test (**a**, **c**) or two-tailed unpaired Student’s t-test (**b**, **d**–**l**). ****P < 0.0001; ***P < 0.001; **P < 0.01; *P < 0.05; ns, not significant. Source data

To determine whether an elevated NAD⁺/NADH ratio contributes to the increased CRC motility, we modulated this ratio. In SORD KO cells, we raised the NAD⁺/NADH ratio using α-ketobutyrate, an exogenous electron acceptor; nicotinamide, an NAD^+^ precursor; or *Lactobacillus brevis* water-forming NADH oxidase (*Lb*NOX)^20–22^ (Extended Data Fig. 6a,b). The increased redox ratio by each approach rescued CRC migration in SORD KO cells (Fig. 4d,e and Extended Data Fig. 6c,d). Conversely, lowering the NAD⁺/NADH ratio in SORD WT cells using FK866 (NAMPT inhibitor) or metformin reduced their migration (Extended Data Fig. 6e–k). FK866 also decreased liver metastasis in Glu + Fru-fed mice injected with SORD WT cells (Extended Data Fig. 6l). Together, these results support a causal role for elevated NAD⁺/NADH in promoting CRC migration and metastasis under Glu + Fru conditions.

### SORD-mediated NAD⁺ regeneration activates the mevalonate pathway to promote CRC migration

Next, we investigated how the increased NAD^+^/NADH ratio contributes to CRC motility. Cancer cells rely on aerobic glycolysis, known as the Warburg effect^23,24^. High glycolytic rates are constrained by the need to replenish cytosolic NAD⁺, as GAPDH consumes NAD⁺ and produces NADH^25^. Aside from two NADH shuttles that regenerate cytosolic NAD^+^ by the tricarboxylic acid (TCA) cycle and mitochondrial respiration, lactate dehydrogenase is the primary mechanism known for rapidly recycling the cytosolic NAD^+^ pool; it directly converts NADH to NAD^+^ in the cytosol by reducing pyruvate to lactate, which is excreted (Fig. 4f). Recent studies suggest that aerobic glycolysis in proliferating cells, such as cancer cells, may be driven more by NAD⁺ demand than by ATP needs^26–29^. We reasoned that NAD^+^ generated by the SORD reverse reaction under Glu + Fru conditions might provide an alternative NAD⁺ source for glycolysis, supplementing lactate dehydrogenase. This may relieve CRC cells of NAD⁺ limitation while preserving glucose carbon in pyruvate and downstream metabolites like acetyl-CoA and TCA intermediates (Fig. 4f). As a result, this could accelerate glycolysis and boost metabolite production in glycolysis and the TCA cycle.

**Extended Data Fig. 7:**
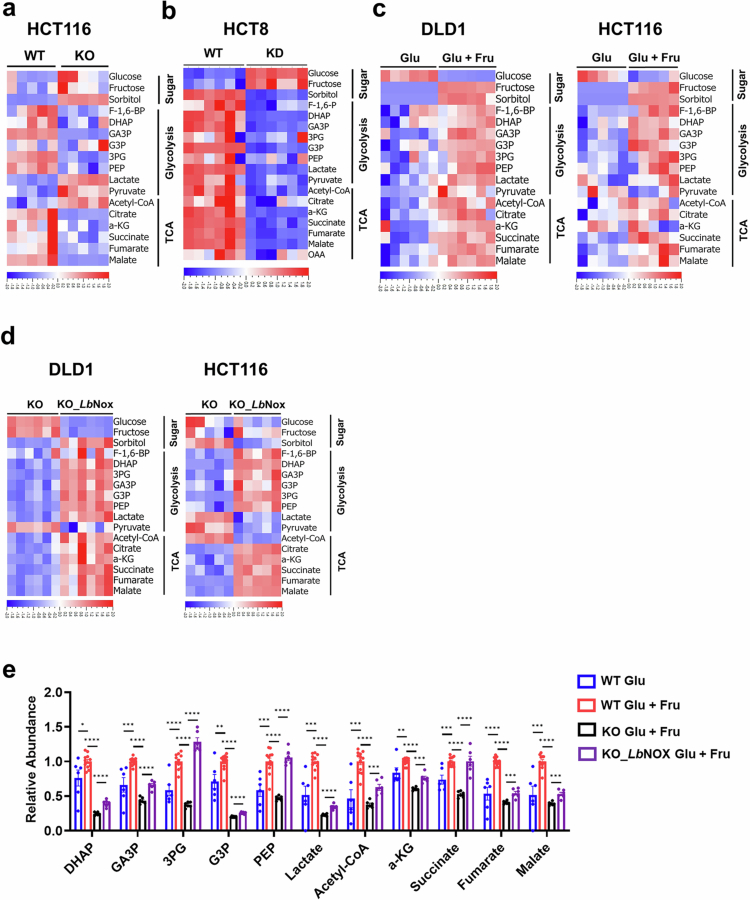
SORD-mediated NAD⁺ regeneration under glucose + fructose conditions activates glycolysis and the TCA cycle. **a**, Heatmap of relative metabolite levels in sugars, glycolysis, and the TCA cycle in SORD WT and KO HCT116 cells after 48 h incubation under Glu + Fru conditions. **b**, Heatmap for SORD WT and Knockdown (KD) cells in HCT8 after 48 hours incubation under Glu + Fru conditions. **c**, Heatmap showing relative metabolite levels in sugars, glycolysis, and the TCA cycle after 48 h treatment under Glu or Glu + Fru conditions in DLD1 (n = 6) and HCT116 (n = 5) cells. **d**, Heatmap of relative metabolite levels in sugars, glycolysis, and the TCA cycle in SORD KO cells and SORD KO cells expressing LbNOX in DLD1 (n = 6) and HCT116 (n = 5) after 48 h under Glu + Fru conditions. **e**, Bar graph showing relative levels of glycolytic and TCA cycle metabolites in DLD1 cells from four groups: SORD WT under Glu (n = 6), SORD WT under Glu + Fru (n = 12), SORD KO under Glu + Fru (n = 6), and SORD KO expressing LbNOX under Glu + Fru (n = 6). Metabolite levels were normalized to SORD WT under Glu + Fru conditions. Medium conditions: Glu, 10 mM glucose; Glu + Fru, 10 mM glucose + 10 mM fructose. Red indicates an increase and blue a decrease in heatmaps (**a**–**d**). All metabolite levels were measured by LC/MS (**a**–**e**). Data represent mean ± s.e.m. from biological replicates. Statistical comparisons were made using a two-tailed unpaired Student’s t-test (**e**). ****P < 0.0001; ***P < 0.001; **P < 0.01; *P < 0.05. F-1,6-BP, fructose-1,6-bisphosphate; DHAP, dihydroxyacetone phosphate; GA3P, glyceraldehyde 3-phosphate; 3PG, 3-phosphoglycerate; G3P, glycerol-3-phosphate; PEP, phosphoenolpyruvate; α-KG, α-ketoglutarate; OAA, oxaloacetate. Source data

To test this idea, we measured fractional labelling of glycolytic and TCA metabolites after exposing SORD WT and KO cells to ^13^C-glucose and unlabelled fructose. As expected, ^13^C incorporation into glycolytic and TCA metabolites was higher in SORD WT than KO cells (Fig. 4g). Similarly, the relative abundance of these metabolites was elevated in SORD WT cells compared to SORD KO cells (Fig. 4h and Extended Data Fig. 7a,b). Importantly, under Glu + Fru conditions, the levels of glycolytic and TCA cycle metabolites increased compared to those observed under glucose-only conditions (Extended Data Fig. 7c). Notably, expression of *Lb*NOX in SORD KO cells restored the levels of these metabolites, suggesting that NAD⁺/NADH restoration activates glycolysis and the TCA cycle (Extended Data Fig. 7d,e). These findings collectively demonstrate that SORD activation under Glu + Fru conditions enhances glycolysis and TCA activity through NAD⁺/NADH modulation.

**Extended Data Fig. 8:**
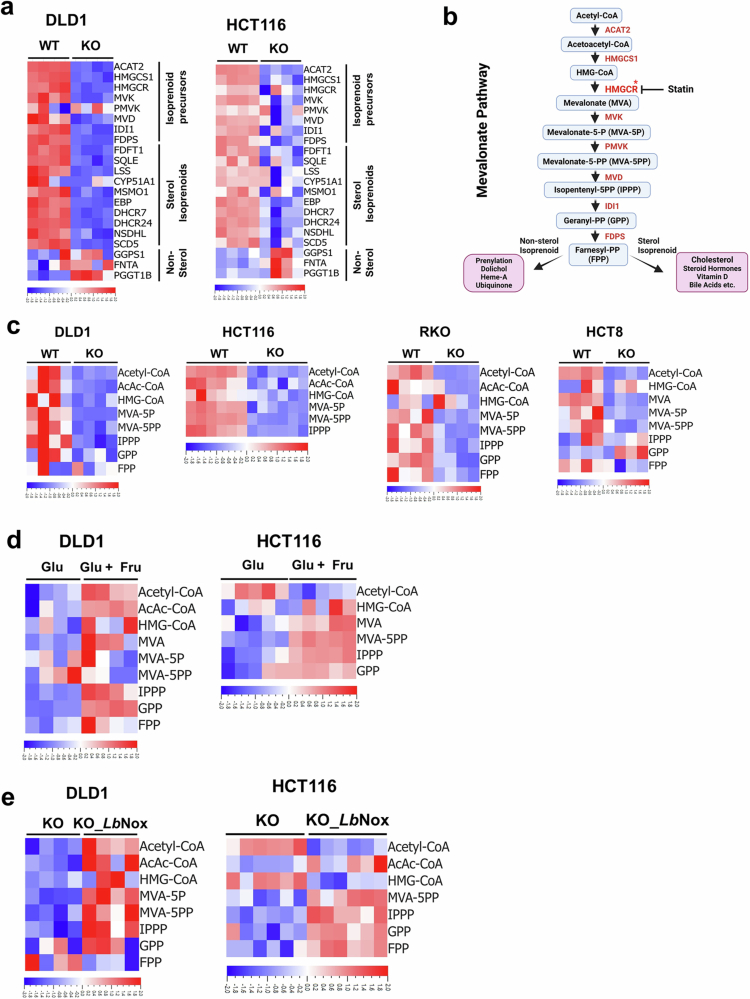
SORD-mediated NAD⁺ regeneration under glucose + fructose conditions activates the mevalonate pathway. **a**, Heatmap of gene expression levels for genes in the mevalonate pathway in SORD WT and KO cells derived from HCT116, based on RNA-seq data. Cells were incubated in Glu + Fru (10 mM each) for 48 h (n = 4). **b**, Schematic of the mevalonate pathway highlighting key enzymes (in red) and metabolites. An asterisk marks HMG-CoA reductase (HMGCR), the pathway’s rate-limiting enzyme. Statins inhibit HMGCR. **c**, Heatmap of relative metabolite levels in the mevalonate pathway in WT and SORD KO cells after 24 h (DLD1 and RKO), 36 h (HCT8), or 48 h (HCT116) under Glu + Fru conditions (DLD1, n = 4; HCT116, n = 6; RKO, n = 4; HCT8, n = 4). **d**, Heatmap of relative metabolite levels in the mevalonate pathway in DLD1 (n = 4) and HCT116 (n = 5) cells after 24 h treatment with Glu (10 mM) or Glu + Fru (10 mM each). **e**, Heatmap of relative metabolite levels in the mevalonate pathway in SORD KO and SORD KO + LbNOX cells from DLD1 (n = 4) and HCT116 (n = 6) after 48 h under Glu + Fru conditions. Red indicates an increase and blue a decrease in all heatmaps. All metabolites were measured using LC–MS. Metabolite abbreviations: AcAc-CoA, acetoacetyl-CoA; HMG-CoA, 3-hydroxy-3-methylglutaryl-CoA; MVA, mevalonate; MVA-5P, mevalonate-5-phosphate; MVA-5PP, mevalonate-5-pyrophosphate; IPPP, isopentenyl pyrophosphate; GPP, geranyl pyrophosphate; FPP, farnesyl pyrophosphate. Extended Data Fig. 8b was created in BioRender. Yun, J. (2025) https://BioRender.com/2ebrqwu. Source data

To understand how increased glycolysis and TCA cycle activity enhance CRC motility, we performed RNA-seq on isogenic SORD WT and KO cells from DLD1 and HCT116. Pathway analysis showed that SORD WT cells upregulated genes in the mevalonate, cholesterol and geranyl diphosphate biosynthesis pathways—components of the mevalonate pathway—compared to SORD KO cells (Fig. 4i and Extended Data Fig. 8a). The mevalonate pathway, which begins with acetyl-CoA from glycolysis, is an essential and complex metabolic pathway that produces numerous de novo isoprenoids, such as cholesterol, vitamin D and bile acids (sterols), as well as dolichol and ubiquinone (non-sterols) (Extended Data Fig. 8b)^30–32^. Consistent with our RNA-seq results, LC–MS analysis showed elevated mevalonate pathway metabolites in SORD WT compared to KO cells (Fig. 4j and Extended Data Fig. 8c). As expected, Glu + Fru conditions and *Lb*NOX expression also increased mevalonate pathway metabolites (Extended Data Fig. 8d,e), in line with their roles in enhancing the NAD⁺/NADH ratio, glycolysis and migration.

**Extended Data Fig. 9:**
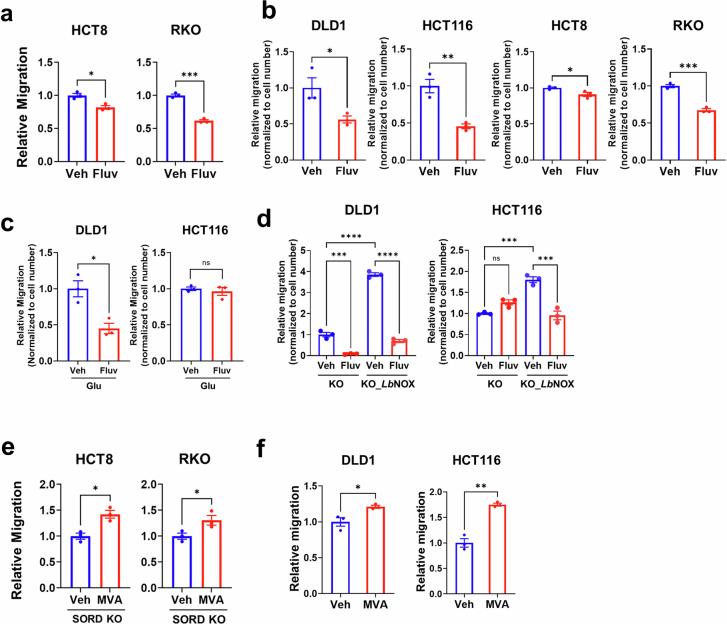
The mevalonate pathway contributes to CRC cell motility. **a**, Migration of SORD WT cells assessed by transwell assay under Glu + Fru conditions with vehicle (Veh) or fluvastatin (Fluv) (n = 3; HCT8, 2 μM; RKO, 0.5 μM). Values were normalized to vehicle. **b**, Normalized migration (by SYBR green assay-based cell number) of SORD WT cells from four CRC cell lines under Glu + Fru condition with vehicle (Veh) or fluvastatin (Fluv) (n = 3; DLD1, 1.5 μM; HCT116, 1 μM; HCT8, 2 μM; RKO, 0.5 μM). Values were normalized to vehicle. **c**, Normalized migration (by SYBR green assay-based cell number) of DLD1 and HCT116 cells under 20 mM glucose (Glu) with vehicle (Veh) or fluvastatin (Fluv) (n = 3). Values were normalized to vehicle. **d**, Normalized migration (by SYBR green assay-based cell number) of SORD KO cells or SORD KO cells expressing LbNOX under Glu + Fru conditions with vehicle (Veh) or fluvastatin (Fluv) (n = 3). Values were normalized to vehicle. **e**, Migration of SORD KO cells treated with vehicle (Veh) or mevalonolactone (MVA; a cell-permeable form of mevalonate) (n = 3; HCT8, 10 mM; RKO, 5 mM). Values were normalized to vehicle. **f**, Transwell migration of SORD WT cells derived from DLD1 and HCT116 treated with vehicle (Veh) or mevalonolactone (MVA; a cell-permeable form of mevalonate) (DLD1, 5 mM; HCT116, 2 mM. n = 3). Values were normalized to the vehicle group. All the in vitro experiments were conducted under Glu + Fru (10 mM each) condition unless otherwise noted. Data represent mean ± s.e.m. from biological replicates. Statistical analysis was performed using two-tailed unpaired Student’s t-test (**a**-**c**, **e**, **f**) or one-way ANOVA with Dunnett’s post hoc test (**d**). ****P < 0.0001; ***P < 0.001; **P < 0.01; *P < 0.05; ns, not significant. Source data

We next investigated the causal role of the mevalonate pathway in CRC migration by treating SORD WT cells with fluvastatin, an HMGCR inhibitor. At doses that did not impair proliferation, HMGCR inhibition significantly suppressed migration in both SORD WT cells and *Lb*Nox-expressing SORD KO cells (Fig. 4k and Extended Data Fig. 9a–d). Conversely, adding cell-permeable mevalonate, a downstream HMGCR product, enhanced migration in both SORD KO and WT cells (Fig. 4l and Extended Data Fig. 9e,f). Oral administration of simvastatin, another statin, significantly reduced liver metastasis in a Glu + Fru-fed caecum injection mouse model (Fig. 4m). These findings indicate that SORD-driven activation of the mevalonate pathway under Glu + Fru conditions promotes CRC migration and metastasis (Fig. 4n).

## Discussion

Despite advances in CRC treatment, metastasis remains the primary cause of CRC-related mortality^17^. Although diet is a key environmental factor in CRC development, the specific dietary components and underlying mechanisms by which diet contributes to metastasis remain unclear, despite prior studies investigating this relationship^33^. Our study fills this gap by establishing a causal link between SSBs and CRC metastasis through SORD. This finding is further supported by human epidemiological studies demonstrating a positive correlation between SSB intake and increased CRC recurrence and mortality^11,12^.

Our findings underscore the need to use both glucose and fructose when studying the effects of SSBs in preclinical models. Prior studies using fructose alone do not reflect the actual composition of SSBs, which contain both sugars^34,35^. Moreover, cells in organisms are never exposed to fructose alone in vivo without glucose, even after consuming 100% fructose solutions—such beverages do not naturally occur. In our study, CRC cells were exposed to a combination of glucose and fructose, closely mimicking the physiological environment resulting from the consumption of SSBs. The Glu + Fru condition enabled the SORD reverse reaction, which was absent under glucose-only or fructose-only conditions. Ultimately, our results revealed that the combination of glucose and fructose uniquely enhances CRC motility and metastasis through SORD-mediated NAD⁺ recycling, supporting enhanced glycolysis and subsequent activation of the mevalonate pathway (Fig. 4n). Although our study demonstrated the direct effects of SSBs on cancer migration and metastasis using cell lines and orthotopic xenografts, further investigation is warranted to elucidate how SSBs influence other components of the tumour microenvironment—such as immune cells and the microbiota—and their collective roles in promoting metastasis.

Our results also suggest potential strategies to inhibit CRC metastasis. First, targeting SORD may reduce CRC metastasis. Historically, research on the polyol pathway has focused primarily on AR in diabetic complications^36,37^, with limited understanding of the role of SORD in cancer^38,39^. Our study showed that SORD deletion suppressed migration even under glucose-only conditions and that SORD is highly expressed in CRC tumours—especially in stem-cell-like tumour cells—together highlighting its fundamental role in CRC motility, independent of sugar conditions. Second, lowering the NAD^+^/NADH ratio in CRC cells may also inhibit metastasis. Recent studies have linked elevated NAD^+^/NADH levels with cell proliferation and survival in various cancers^26–29^. Our findings reveal a previously unappreciated role for elevated NAD⁺/NADH levels in promoting CRC migration and metastasis, independent of their effects on cell proliferation. Third, statins may be repurposed to target CRC metastasis, as our study demonstrates that activation of the mevalonate pathway under Glu + Fru conditions contributes to CRC metastasis. However, the detailed underlying mechanisms—such as how SORD deletion leads to decreased expression of mevalonate pathway genes, and how activation of this pathway promotes metastasis—remain unclear, reflecting the broader complexity of this pathway in cancer metastasis^31,32,40^. Although preclinical studies suggest that statins may serve as effective anti-cancer agents^41^, epidemiological results have been mixed regarding their effects on CRC survival^42,43^. Given variability in SORD expression and dietary sugar intake among patients, statins may be especially beneficial in cases with high SORD expression and high SSB consumption—supporting the rationale for evaluating these links through a prospective randomized cohort. Lastly, our preclinical data highlight the potential of SSB consumption to accelerate CRC metastasis, underscoring the urgent need for dietary guidance focused on reducing SSB intake to lower CRC mortality.

## Methods

### Cell culture and cell lines

The following CRC cell lines were obtained from the American Type Culture Collection (ATCC): HCT116 (ATCC, CCL-247), DLD1 (ATCC, CCL-221), RKO (ATCC, CRL-2577), HCT8 (ATCC, CCL-244), NCI-H508 (ATCC, CCL-253), Colo205 (ATCC, CCL-222), SW620 (ATCC, CCL-227), SW48 (ATCC, CCL-231) and HCT15 (ATCC, CCL-225). The DiFi, GP5D and HT55 cell lines were provided by J. Engelman, and the VACO432 cell line was provided by S. Markowitz. Short tandem repeat profiling was performed to authenticate all cell lines. Cells were cultured in high-glucose DMEM (Fisher Scientific, 11-965-118) supplemented with 10% FBS (Fisher Scientific, 26-140-079). All cultures were maintained at 37 °C in a humidified atmosphere containing 5% CO_2_.

### In vitro sugar experiments

Three media formulations were used for in vitro sugar experiments. The Glu + Fru condition contained 10 mM glucose and 10 mM fructose. The Glu condition contained 20 mM glucose, and the Fru condition contained 20 mM fructose. All media were prepared using glucose-free DMEM (Fisher Scientific, 11-966-025) supplemented with 10% dialysed FBS (Fisher Scientific, SH30079.03). d-Glucose (Sigma-Aldrich, G8270) and d-fructose (Sigma-Aldrich, F0127) were added to achieve the desired concentrations. For isotope-labelling metabolomics, media were prepared by replacing d-glucose with [U-^13^C_6_] d-glucose (Cambridge Isotope Laboratories, CLM-1396-10) or d-fructose with [U-^13^C_6_] d-fructose (Cambridge Isotope Laboratories, CLM-1553-PK).

### Migration and invasion assays using transwell

The migration and invasion assays were performed as previously described^44^. For the migration assay, 100 μl of CRC cells (6 × 10^5^ for DLD1, 8 × 10^5^ for HCT116, 4–5 × 10^5^ for HCT8, 5–8 × 10^5^ for RKO and 5 × 10^5^ for the remaining nine CRC cell lines) in serum-free assay medium was seeded into the cell culture insert (upper chamber) of a transwell (Fisher Scientific, 07-200-150). The lower chamber was filled with 600 μl of assay medium containing 10% dialysed FBS (Fisher Scientific, SH30079.03). For chemical treatments, compounds were added to both the upper and lower chambers. After incubation for 48–72 h (48–72 h for DLD1 and HCT116, 48 h for HCT8, 60–72 h for RKO and 48 h for the remaining nine CRC cell lines), the bottom surfaces of the inserts were stained with 0.1% crystal violet (Sigma-Aldrich, C0775) in 20% methanol for 10 min. Non-migrated cells on the upper side of the membrane were removed with cotton swabs before analysis.

For the invasion assay, 40 μl of 15% Matrigel (Fisher Scientific, CB40230C) in serum-free assay medium was added to the insert, and the plate was incubated at 37 °C for 30 min before cell seeding. Cells were seeded using the same numbers as in the migration assay, and the same assay medium was added. The incubation time for the invasion assay was 24–48 h longer than for the migration assay. After incubation, the bottom surfaces of the inserts were stained with 0.1% crystal violet (Sigma-Aldrich, C0775) in 20% methanol for 10 min, and non-invaded cells on the upper side of the membrane were removed with cotton swabs before analysis.

Unless otherwise specified, the assay medium used in both migration and invasion assays was glucose-free DMEM supplemented with either 10 mM glucose and 10 mM fructose (Glu + Fru condition) or 20 mM glucose (Glu condition), and 10% dialysed FBS. The compounds used in these assays included fluvastatin (Sigma-Aldrich, PHR1620-1G), FK866 (APExBIO, A4381), (±)-mevalonolactone (a cell-permeable form of mevalonate; Sigma-Aldrich, M4667-1G), metformin (Cayman Chemical, 13118), α-ketobutyrate (Sigma-Aldrich, K401-5G) and nicotinamide (Sigma-Aldrich, N0636). All chemical doses used for migration assays were optimized to minimize cytotoxicity, and cell numbers were measured in parallel using the SYBR Green assay under the same treatment conditions. Although nicotinamide and mevalonolactone did not induce cytotoxicity, FK866, metformin and fluvastatin showed mild cytotoxic effects in certain cell lines, even at optimized concentrations. Therefore, migration data were normalized to cell number to account for potential proliferation differences, as presented in the Extended Data. Significant suppression of migration persisted after normalization, confirming that reduced motility was not solely caused by effects on cell viability. After drying the membranes, images were acquired by microscopy and quantified by measuring the stained area using ImageJ (v.1.54 f).

### Cell growth and viability assays

For the growth assay, CRC cells were seeded into 96-well plates (4,000 cells per well) and cultured for 1–5 days. After incubation, either the SYBR Green I assay or CellTiter-Glo Luminescent Cell Viability Assay (Promega, G7571) was used to quantify cell growth, depending on the experiment. For the SYBR Green I assay, the medium was removed and 50 μl of 0.2% SDS was added to each well, followed by 150 μl of diluted SYBR Green I solution (1:750 in water) (Fisher Scientific, S-7567). Fluorescence was measured using a plate reader (BioTek, Synergy H1) at excitation 485 nm and emission 528 nm. For experiments using CellTiter-Glo, the assay was performed according to the manufacturer’s instructions.

For cell viability measurements in transwell-based migration or invasion assays, the same number of cells used in the transwell inserts was seeded into 24-well plates. Cells were cultured under identical conditions (100 μl serum-free assay medium and 600 μl assay medium with 10% dialysed FBS) and for the same durations as the corresponding migration or invasion assays. After incubation, 200 μl of 0.2% SDS was added to each well. Cell lysates were sonicated for five cycles (2 s on, 2 s off, 30% amplitude) using a probe sonicator (Active Motif EpiShear, Q120AM) to homogenize the lysate. Then, 50 μl of the lysate was transferred to a new well, and 150 μl of diluted SYBR Green I solution (1:750 in water) was added. Fluorescence was measured as described above using the same plate reader.

### Colony formation assay

The clonogenic ability of CRC cells was measured using a previously described protocol^45^. In brief, 500–2,000 CRC cells per well were seeded into six-well plates under Glu + Fru or Glu conditions and cultured for 10–14 days. The medium was then removed, and each well was washed twice with 2 ml of 1× PBS. Cells were fixed with 400 μl of methanol at room temperature (20–25 °C) for 20 min. After removing the methanol, 1 ml of 0.5% crystal violet (Sigma-Aldrich, C0775) in 25% methanol was added to each well and incubated for 40 min. The stain was then removed, and the wells were washed with deionized water. Plates were dried overnight at room temperature, and individual colonies in each well were manually counted after image acquisition and marking using ImageJ (v.1.54f).

### Laboratory animals and sugar treatments

Male NU/J (athymic) mice (7 weeks old ) were purchased from The Jackson Laboratory (strain no. 002019), housed in a modified barrier facility and used for caecum orthotopic injection of CRC cells. Male and female NOD scid gamma (NSG) mice were purchased from The Jackson Laboratory (strain no. 005557) and housed in a high-barrier facility optimized for immunodeficient mice. Parental and F1 generation NSG mice were used for breeding, and F1 and F2 generation male mice were used for colon mucosal and splenic injection of CRC cells. All mice were housed in a controlled environment (22 ± 1 °C, 60–70% humidity, 12 h light–dark cycle) and fed ad libitum with standard chow (Picolab Rodent Diet 20, 5053).

For sugar treatment experiments, mice were given ad libitum access to either tap water, glucose solution (100 g glucose in 400 ml autoclaved tap water; 25% w/v) or Glu + Fru solution (45 g glucose + 55 g fructose in 400 ml autoclaved tap water; 25% w/v). These special water bottles were changed twice per week. In separate studies, mice received daily oral gavage with Glu + Fru (45 mg glucose + 55 mg fructose in 400 μl tap water), glucose alone (100 mg in 400 μl tap water) or tap water alone (400 μl). All tap water used for mouse treatments was autoclaved.

All animal experiments were approved by the Institutional Animal Care and Use Committees (IACUC) of Baylor College of Medicine (BCM) and the MD Anderson Cancer Center. The maximal tumour burden permitted by the IACUC (≤5 mm diameter for oral, head, neck and osseous tumours) was not exceeded in this study.

### Caecum injection mouse model

The caecum orthotopic injection of CRC cells into the NU/J mice was conducted as previously described^46^. Male NU/J mice (8 weeks old) with similar body weights were randomly assigned to experimental groups. Before surgery, mice were administered preemptive analgesia with buprenorphine ER (ZooPharm LLC, Rx 255459) and meloxicam (Covetrus, 11695-6936-1), and anaesthetized with 3% isoflurane (Covetrus, 11695-6777-2) in 2 l min^−1^ oxygen. The abdominal surgical site was sterilized, and a 1–2 cm incision was made in the skin and abdominal wall musculature to expose the caecum. A single-cell suspension of 2 × 10^6^ CRC cells in 30 μl of 100% prechilled Matrigel (Corning, CB40230C) was carefully injected into the caecal wall using 29-gauge insulin syringes (BD Medical, 324702). Excess tumour cells were gently removed using saline-moistened sterile gauze. The caecum was returned to the abdominal cavity, and the incision was closed using absorbable polyglycolic acid 4-0 suture (Ethicon, J303H) and wound clips (CellPoint Scientific, 201-1000). Analgesic drugs were administered for 3 days postoperatively, and wound clips were removed after 10 days. Body weight and food and water consumption were recorded weekly. After 4–6 weeks, all mice from the same cohort were killed on the same date, and necropsies were performed to examine tumour metastasis and collect tissues.

### Intrasplenic injection mouse model

Intrasplenic injection of CRC cells was conducted following the previously described protocol^47^. NSG mice (8 weeks old) with similar body weights were randomly assigned to experimental groups. Before surgery, mice were administered preemptive analgesia with buprenorphine ER (ZooPharm LLC, Rx 255459) and meloxicam (Covetrus, 11695-6936-1), and anaesthetized with 3% isoflurane (Covetrus, 11695-6777-2) in 2 l min^−1^ oxygen. The left abdominal side, previously shaved, was sterilized using three alternating scrubs with 5% povidone–iodine solution (Veterinary Betadine, 67618-154-16) and alcohol prep pads (Covidien, 6818). A 1 cm incision was made in the skin and abdominal wall musculature at the estimated location of the spleen. The spleen was exposed and gently ligated with absorbable polyglycolic acid 4-0 suture. A single-cell suspension of 7 × 10^5^ CRC cells in 30 μl of PBS was injected into the spleen using a 30-gauge syringe (BD, 305106). The spleen was then isolated with the suture and removed using a low-temperature cautery (World Precision Instruments, 500390). The abdominal wall was closed with absorbable suture (Ethicon, J303H), and the skin was closed with wound clips (CellPoint Scientific, 201-1000). Analgesic drugs were administered for 3 days postoperatively, and wound clips were removed after 10 days. Body weight and food and water consumption were recorded weekly. The Lumina In Vivo Imaging System (IVIS) was used to monitor the bioluminescence of labelled cells each week. After 4–6 weeks, all mice from the same cohort were killed on the same date, and necropsies were performed to examine tumour metastasis and collect tissues.

### Colonic mucosal injection mouse model

The colonoscopy-based colonic mucosal injection of CRC cells was performed as previously described^18^. First, the colonoscopy system—consisting of a miniature endoscope (1.9 mm outer diameter), a xenon light source, a triple-chip camera and an air pump (Karl Storz, Tuttlingen, Germany)—was set up. NSG mice (8 weeks old) with similar body weights were randomly assigned to experimental groups. Before the experiment, mice were anaesthetized using 3% isoflurane (Covetrus, 11695-6777-2) in 2 l min^−1^ oxygen, and the colonoscope was inserted about 2 cm into the rectum. A sterile 30-gauge needle attached to plastic tubing (provided with the colonoscope) was inserted into the working channel, and the needle was advanced into the colonic wall at a 45-degree angle. A single-cell suspension of 1 × 10^5^ CRC cells in 40 μl of PBS was injected into the colonic wall (20 μl per site, two sites per mouse). Body weight and food and water consumption were recorded weekly. Colonoscopy was used weekly to visualize colonic tumours after cell implantation. After 4 weeks, all mice from the same cohort were killed on the same date, and necropsies were performed to examine tumour metastasis and collect tissues.

### Measurement of in vivo luminescence in mice using IVIS

Mice implanted with luciferase-expressing cancer cells were intraperitoneally injected with 100 μl of 15 mg ml^−1^ luciferin (Gold Biotechnology, LUCK-1G) per mouse. Then, 7 min later, mice were anaesthetized using the XGI-8 Gas Anesthesia System (PerkinElmer, XGI-8) with 3% isoflurane (Covetrus, 11695-6777-2) in 2 l min^−1^ oxygen. Then, 10 min after luciferin injection, mice were transferred to the Lumina IVIS (PerkinElmer). Images with exposure times of 1 s, 5 s, 10 s, 30 s, 1 min and 2 min were acquired according to the equipment manual and used for further analysis. For liver imaging, 10 min after luciferin injection, mice were killed using CO_2_, and livers were immediately collected and placed in the Lumina IVIS. Images with exposure times of 2 s, 5 s and 10 s were recorded and used for further analysis. Total luminescence values were quantified using Aura Software (Spectral Instruments Imaging, Aura Software, v.4.0.7).

### Drug treatments of mice

In the FK866 treatment experiment, mice were treated with 10 mg kg^−1^ FK866 (APExBIO, A4381) in PBS (Corning, MT 21-040-CV) containing 2% dimethylsulfoxide (DMSO) (Sigma-Aldrich, D4540) by intraperitoneal injection. In the simvastatin treatment experiment, mice were treated with 30 mg kg^−1^ day^−1^ simvastatin (Selleckchem, S1792) in PBS containing 10% DMSO by oral gavage. During the drug treatment period, mice had ad libitum access to the Glu + Fru solution (25% w/v, 45:55 ratio) in their drinking water.

### H&E and immunohistochemistry staining

Mouse tissues were placed in tissue cassettes (Thermo, 22-272417) and fixed in 4% paraformaldehyde in PBS (Santa Cruz, sc-281692) for 24 h at 4 °C. Tissues were then transferred to 70% ethanol (VWR, 89125-188) before processing. Tissue processing, embedding, sectioning and H&E staining were performed by the BCM Breast Cancer Histology Core at BCM. Stained slides were scanned by HistoWiz for bright-field whole-slide scanning.

Human tissue slides for H&E and SORD immunohistochemistry (IHC) staining were obtained from the Human Tissue Acquisition and Pathology (HTAP) Services at BCM. All original tumour tissues and matched normal tissues (not all samples included matched normal tissues) were collected at the Michael E. DeBakey Veterans Affairs Hospital, Ben Taub General Hospital and Baylor St. Luke’s Medical Center. Tissue acquisition was based on a system designed to maximize the number of tissues available from BCM-affiliated institutions. The process began with a patient tissue advocate who identified all tissues with the potential to be banked for HTAP each day. A redundant consenting protocol, which included multiple attempts to obtain patient consent, was used. After collection, tissues were barcoded, organized, catalogued and banked in the tissue database. H&E and IHC staining of human tissues were performed by the BCM Breast Cancer Histology Core. The SORD antibody (Sigma-Aldrich, HPA040621) used for IHC staining was validated on SORD KO and SORD-proficient xenograft mouse tissues before application on human slides to ensure antibody specificity. Stained slides were scanned by HistoWiz for bright-field whole-slide scanning.

### Human colon tumour organoid and normal colon organoid culture

Human colon tumour organoids were provided by M. L. Martin at Weill Cornell Medicine (the Englander Institute for Precision Medicine), and human normal colon organoids (also called enteroids) were acquired from BCM (Gastrointestinal Experimental Model Systems Core). All 3D cultures of organoids were maintained in 15 μl per drop, totalling five drops (75 μl per well) of Matrigel (Gibco, CB40230C) in 12-well plates (Life Technologies, 150628) with 1 ml of the following medium in a 37 °C, 5% CO_2_ humidified incubator. The medium was changed every 2 days, and organoids and enteroids were passaged at a 1:6 ratio when crowded.

The medium for culturing human colon tumour organoids contained the following components: Advanced DMEM/F12 (Gibco, 12634010), B27 Supplement (Invitrogen, 17504001) at 1× concentration, l-glutamine (Gibco, 25030164) at 2 mM, HEPES (Amresco, J848) at 10 mM, Primocin (Invitrogen, NC9141851) at 100 μg ml^−1^, human recombinant EGF (R&D Systems, 236-EG-200) at 50 ng ml^−1^, Gastrin I (Sigma-Aldrich, SCP0152) at 10 nM, Y-27632 (cAMP inhibitor; Selleck Chemicals, S1049) at 10 μM, A-83-01 (TGF-β inhibitor; R&D Systems, 2939) at 500 nM, SB202190 (MAPK inhibitor; Selleck Chemicals, S1077) at 3 μM, nicotinamide (Sigma-Aldrich, N3376) at 10 mM, *N*-acetylcysteine (Sigma-Aldrich, A9165) at 1.25 mM, prostaglandin E_2_ (Sigma-Aldrich, P0409) at 10 nM, Noggin (10% conditioned medium), R-spondin (10% conditioned medium) and penicillin–streptomycin (Gibco, 15140-122) at 1× concentration.

The medium for culturing human colon organoids contained the following components: Advanced DMEM/F12 (Gibco, 12634010), B27 Supplement (Invitrogen, 17504001) at 1× concentration, l-glutamine (Gibco, 25030164) at 2 mM, HEPES (Amresco, J848) at 10 mM, Primocin (Invitrogen, NC9141851) at 100 μg ml^−1^, human recombinant EGF (R&D Systems, 236-EG-200) at 50 ng ml^−1^, Gastrin I (Sigma-Aldrich, SCP0152) at 10 nM, Y-27632 (cAMP inhibitor; Selleck Chemicals, S1049) at 10 μM, A-83-01 (TGF-β inhibitor; R&D Systems, 2939) at 500 nM, SB202190 (MAPK inhibitor; Selleck Chemicals, S1077) at 10 μM, nicotinamide (Sigma-Aldrich, N3376) at 10 mM, *N*-acetylcysteine (Sigma-Aldrich, A9165) at 1 mM, prostaglandin E_2_ (Sigma-Aldrich, P0409) at 10 nM, penicillin–streptomycin (Gibco, 15140-122) at 1× concentration, Noggin conditioned medium (10%), R-spondin conditioned medium (10%) and WNT-3A conditioned medium (30%).

### Human enteroid 2D monolayer differentiation

The collagen IV stock solution was prepared by dissolving collagen IV (1 mg ml^−1^; Sigma-Aldrich, C5533-5MG) in 100 mM acetic acid (0.6% acetic acid in H_2_O). A 24-well plate was coated with 500 μl per well of the collagen working solution (prepared by diluting the collagen IV stock 1:30 (v/v) in ice-cold water) and incubated at 37 °C for 1.5 h. Three-dimensional cultured enteroids were dissociated using 500 μl per well of 0.5 mM EDTA (Fisher, BP24821) diluted in PBS. Enteroids from one well of a 12-well plate (approximately 75 μl) were used to seed one well of a 24-well plate. After centrifugation at 300*g* at 4 °C for 5 min, the pellet was resuspended in 500 μl of 0.05% trypsin and 0.5 mM EDTA and incubated at 37 °C for 5 min. Then, 1 ml of DMEM/F12 supplemented with 10% FBS was added, and the mixture was pipetted 60 times to dissociate the cells. Following centrifugation at 400*g* at 4 °C for 5 min, the enteroid pellet was resuspended in 500 μl of enteroid medium and seeded onto the coated plate. After 1 day, the medium was replaced with differentiation medium lacking R-spondin, WNT-3A, nicotinamide and SB202190, and the percentage of Noggin conditioned medium was reduced to 5%. The medium was refreshed every 2 days.

### Reverse transcription and qPCR

RNA from organoids, enteroids and 2D-differentiated enteroids was extracted using the RNeasy Plus Mini Kit (Qiagen, 74136), and cDNA was synthesized using the SuperScript IV VILO Kit (Fisher, 11756050) following the manufacturer’s instructions. Real-time qPCR was performed using amfiSure qGreen Q-PCR Master Mix (GenDEPOT, Q5600-010) on a CFX96 Real-Time System (Bio-Rad). Primers for qPCR included ACTB1 F, GCAAAGACCTGTACGCCAAC; ACTB1 R, ACATCTGCTGGAAGGTGGAC; SORD F, GGCTCTGAGATGACCACCGT; SORD R, GGTCACACTTGAGCATGATTTTCA; LGR5 F, CCTGCTTGACTTTGAGGAAGACC; and LGR5 R, CCAGCCATCAAGCAGGTGTTCA. All primers were ordered from Thermo Fisher Scientific.

### Plasmids for gene KO and knockdown of CRC cell lines and *Lb*NOX

CRISPR–Cas9-mediated genome editing was used to generate SORD KO cell lines^48^. The gRNA sequences (sgSORD-1, AGCAAAGTGACCATCCCGAT; sgSORD-2, TTGTTTAGGGCCAATCGGGA; sgAKR1B1-1, TCAGGTCGCTGAGTGTCTTC; sgAKR1B1-2, TCCCATACCTTAAAGCCAGT) were cloned into the lentiCRISPRv2-puro plasmid (Addgene, 98290) using the restriction enzyme BsmBI (Thermo, FD0454). The plasmids were then packaged into lentivirus and transduced into CRC cell lines.

Pre-designed short hairpin RNA (shRNA) plasmids for SORD knockdown—including SORD shRNA1 (TRC Clone ID, TRCN0000028069; target sequence, GAGAACTATCCTATCCCTGAA), SORD shRNA2 (TRC Clone ID, TRCN0000028052; target sequence, GCCAATCGGGATGGTCACTTT) and SORD shRNA3 (TRC Clone ID, TRCN0000028106; target sequence, CGTCCAAGTCTGTGAATGTAA)—were purchased from MilliporeSigma. These plasmids were also packaged into lentivirus and transduced into CRC cell lines.

The *Lb*NOX open reading frame was amplified from pUC57-*Lb*NOX (Addgene, 75285) by PCR^20,21^ using the following primers: F, TCTAGAATGAAGGTCACCGTGGTCGG; R, GTTCGAATTACTTGTCATCGTCATCCTTGTAA. The open reading frame was then cloned into the pCDH-EF1-MCS-IRES-copGFP plasmid (System Biosciences, CD530A-2) for *Lb*NOX overexpression. Plasmids were transfected into cell lines using Lipofectamine (Invitrogen, 100022050 and 100022057). GFP-positive cells were sorted by flow cytometry (Beckman, CytoFLEX SRT) and used for subsequent experiments.

### Lentivirus package

Plasmids were co-transfected with d8.2 (Addgene, 8455) and VSVG (Addgene, 8454) at a ratio of 1:1:0.1 into HEK293T cells using threefold (m/m) polyethylenimine (Sigma-Aldrich, 764965). After 2 days of culture at 37 °C with 5% CO_2_, the medium was collected, filtered through a 0.45 μm filter (Millipore, SLHVM33RS) and stored at −80 °C before use. HCT116, DLD1, HCT8 and RKO CRC cell lines were infected with the packaged lentivirus, and positive cells were selected with puromycin (InvivoGen, ant-pr-5) at the following concentrations: 2 μg ml^−1^ for DLD1, 1 μg ml^−1^ for HCT116, 15 μg ml^−1^ for HCT8 and 1 μg ml^−1^ for RKO.

### Immunoblotting

Proteins were extracted from tissues or cells using RIPA buffer (Cell Signaling Technology, 9806S) containing a protease inhibitor cocktail (GenDEPOT, P3100-001), separated on 4–20% Criterion TGX Stain-Free Gels (Bio-Rad, 5678094 and 5678095) and transferred to 0.45 μm PVDF membranes (MilliporeSigma, IPVH00010). Membranes were probed with primary antibodies at 4 °C overnight and incubated with horseradish peroxidase (HRP)-conjugated secondary antibodies at room temperature for 1 h. Protein bands were visualized using ECL western blotting substrates (Bio-Rad, 1705060 and 1705062) and imaged using the ChemiDoc Imaging System (Bio-Rad).

The antibodies used included SORD (1:1,000; Proteintech, 15881-1-AP), AKR1B1 (1:1,000; Proteintech, 15439-1-AP), β-actin (1:2,000; Cell Signaling Technology, 3700), goat anti-rabbit IgG(H+L)-HRP (1:5,000; GenDEPOT, SA002) and goat anti-mouse IgG(H+L)-HRP (1:5,000, GenDEPOT, SA001).

### NAD⁺/NADH ratio measurement by enzymatic assay

Cells were plated in six-well dishes with standard DMEM + 10% FBS and cultured overnight. Treatments were applied the following day and continued for 24 h. Cells were then washed with PBS and extracted in 200 μl of ice-cold lysis buffer (1% dodecyltrimethylammonium bromide (Sigma-Aldrich, D8638) in 0.2 N NaOH), diluted 1:1 with PBS and centrifuged at 15,000*g* for 5 min. For NADH measurement, 20 μl of lysate was transferred to PCR tubes and incubated at 75 °C for 30 min. For NAD⁺ measurement, 20 μl of lysate was transferred to PCR tubes, then 20 μl of lysis buffer and 20 μl of 0.4 N HCl were added, followed by incubation at 60 °C for 20 min. After the respective incubations, samples were cooled to room temperature and quenched with the appropriate neutralizing solution: 20 μl of 0.25 M Tris in 0.2 N HCl for NADH and 20 μl of 0.5 M Tris base for NAD⁺. Following sample preparation, enzyme-linked luminescence-based detection of NAD⁺ and NADH was performed according to the manufacturer’s instructions using the NAD⁺/NADH-Glo Assay Kit (Promega, G9071).

### Peredox NADH biosensor to measure NAD^+^/NADH ratio

The cytosolic Peredox NADH biosensor plasmid (Addgene, 32383) was purchased from Addgene and transiently transfected into CRC cell lines using Lipofectamine 3000 (Thermo Fisher, L3000008) 24 h before sugar treatment. Transfected cells were incubated under Glu + Fru conditions for 24 h. Green (excitation, 395/25 nm; emission, 525/34 nm) and red (excitation, 555/15 nm, emission, 605/52 nm) fluorescence channels were acquired using the Eclipse Ti2 inverted Nikon microscope equipped with the Photometrics Prime 95B Scientific CMOS monochrome camera, Lumencor Spectra X light engine and OKO Labs micro-incubator, located at the Basic Sciences Research Building Microscopy Core, Department of Genetics, MD Anderson Cancer Center. Fluorescence intensities were quantified using NIS-Elements Imaging Software (v.5.42.03) as previously described^19,49^

### Metabolomics and isotope-labelled metabolomics

Carbon metabolism metabolites, including intermediates from glycolysis and the TCA cycle as well as sugars such as glucose, fructose and sorbitol, were detected by LC–MS using a previously established protocol^50,51^. For isotope-labelling experiments, cells were treated with glucose-free DMEM (Gibco, 11966025) supplemented with either unlabelled d-fructose (Sigma-Aldrich, F0127) and d-glucose (Sigma-Aldrich, G8270) or ^13^C_6_ d-fructose (Cambridge Isotope Laboratories, CLM-1553-PK) and ^13^C_6_ d-glucose (Cambridge Isotope Laboratories, CLM-1396-10). Cell extracts were prepared by adding 400 μl of 40:40:20 acetonitrile (ACN):methanol:water containing 0.5% formic acid to each well of a six-well plate, followed by neutralization with 35.2 μl of 15% ammonium bicarbonate. After centrifugation at 20,000*g* for 15 min at 4 °C, the supernatants were transferred to sample vials (Thermo, 2-SVWGK) for LC–MS analysis. High-performance LC was performed using an XBridge BEH Amide XP Column (Waters, 186006724). Mobile phase A consisted of 5% ACN with 20 mM ammonium acetate and 20 mM ammonium hydroxide (pH 9.75–9.85); mobile phase B was 100% ACN. The column temperature was maintained at 25 °C, and the flow rate was 0.15 ml min^−1^. The LC gradient was as follows: 0 min, 85% B; 2 min, 85% B; 3 min, 80% B; 5 min, 80% B; 6 min, 75% B; 7 min, 75% B; 8 min, 70% B; 9 min, 70% B; 10 min, 50% B; 12 min, 50% B; 13 min, 25% B; 16 min, 25% B; 18 min, 0% B; 23 min, 0% B; 24 min, 85% B; and 30 min, 85% B. MS was performed on a Q Exactive Orbitrap Mass Spectrometer (Thermo) in both positive and negative ion modes. Data were processed and analysed using TraceFinder software (Thermo, OPTON-31001).

Untargeted global metabolomics analysis using LC–tandem MS (MS/MS), measuring more than 700 metabolites, was conducted as previously described^9^. Specifically, 600 μl of −20 °C 3 mM antioxidant monobromobimane (Fisher Scientific, M20381) in methanol (80:20, v/v) was added to cell samples and incubated for 2 h at room temperature, followed by 1 h of incubation at 0 °C. Samples were then centrifuged for 15 min at 16,000*g*, and supernatants were transferred to clean tubes. This extraction step was repeated two additional times, and the combined supernatants were dried using a SpeedVac (Savant) and stored at −80 °C until analysis. For normalization, post-extraction tissue or tumour pellets were solubilized in 800 μl of 0.2 M aqueous NaOH at 95 °C for 60 min, and protein content was quantified using the BCA Assay (Thermo, PI23227). For metabolite analysis, dried extracts were reconstituted in ACN (70:30, v/v) containing 0.025% acetic acid, and 3 μl of the solution was injected for LC–MS. Metabolite profiling was performed using an Agilent 1200 LC system coupled to an Agilent 6230 time-of-flight mass analyser. Chromatographic separation was achieved using aqueous normal-phase gradient separation on a Diamond Hydride column (Microsolv). Mobile phase A consisted of 6 μM EDTA and 0.025% acetic acid in isopropanol (50:50, v/v); mobile phase B consisted of 6 μM EDTA and 5 mM ammonium acetate in ACN (90:10, v/v). The following LC gradient was applied: 0–1.0 min, 99% B; 1.0–15.0 min, 20% B; 15.1–29.0 min, 0% B; and 29.1–37.0 min, 99% B. Both positive and negative ion mass spectra were acquired in 2 GHz (extended dynamic range) mode at 1.41 spectra per second over a mass/charge range of 40–1400 *m*/*z*. Data were saved in both centroid and profile modes using Agilent MassHunter Workstation B.06.00 Data Acquisition Software. Raw data files were analysed using Mass Profiler Professional (Agilent, v.B.14.5) and MassHunter Profinder (Agilent, v.B.08.00).

For targeted metabolomics, cell lysates were extracted twice with 500 μl of 80% ice-cold methanol. After centrifugation at 20,000*g* for 20 min at 4 °C, the supernatants were dried and reconstituted in 15 μl of LC–MS-grade water, then transferred to sample vials (Thermo, 2-SVWGK). LC–MS/MS and data analysis for untargeted polar metabolomics were performed by the BIDMC Mass Spectrometry Core Facility at Beth Israel Deaconess Medical Center, as previously described^52^. Absolute quantification of mevalonate pathway metabolites was performed by Creative Proteomics using LC–MS. In brief, samples were extracted with 80% methanol as described above, reconstituted in 20 μl of high-performance LC-grade water and 5–7 μl was injected into a hybrid 6500 QTRAP triple quadrupole mass spectrometer (AB SCIEX) coupled to a Prominence UFLC HPLC system (Shimadzu). Metabolites were analysed by selected reaction monitoring (SRM), targeting a panel of 300 endogenous water-soluble metabolites. Some metabolites were detected in both positive and negative ion modes, yielding a total of 311 SRM transitions using polarity switching. The electrospray ionization voltage was +4950 V in positive mode and −4,500 V in negative mode. The dwell time was 3 ms per SRM transition, and total cycle time was 1.55 s. Approximately 9–12 data points were acquired per detected metabolite.

For mevalonate pathway metabolite measurements, cells cultured in 10 cm dishes were washed with 5 ml cold PBS and extracted with 1 ml of 80% methanol, then placed on ice for 1 h. The extracts were centrifuged at 16,000*g* for 15 min, and 100 μl of the supernatant was stored for analysis. The remaining 900 μl was dried using a SpeedVac (Eppendorf, Vacufuge) and reconstituted in 80 μl of 80% methanol. For LC–MS analysis, 5 μl of both unconcentrated and concentrated samples was injected. LC was performed using an XBridge BEH Amide XP Column (Waters, 186006724). Mobile phase A consisted of 5% ACN with 20 mM ammonium acetate and 20 mM ammonium hydroxide (pH 9.75–9.85); mobile phase B was 100% ACN. The column was maintained at 25 °C, and the flow rate was 0.15 ml min^−1^. The LC gradient was as follows: 0 min, 85% B; 2 min, 85% B; 3 min, 80% B; 5 min, 80% B; 6 min, 75% B; 7 min, 75% B; 8 min, 70% B; 9 min, 70% B; 10 min, 50% B; 12 min, 50% B; 13 min, 25% B; 16 min, 25% B; 18 min, 0% B; 23 min, 0% B; 24 min, 85% B; and 30 min, 85% B. MS was performed using a Q Exactive Orbitrap Mass Spectrometer (Thermo) in negative ion mode. Data were processed and analysed using TraceFinder software (Thermo, OPTON-31001).

For the measurement of sorbitol in both cells and medium at different time points, cells were cultured in six-well plates under the indicated conditions, as shown in the figures. At the end of treatment, 1 ml of medium was mixed with 3 ml of methanol and centrifuged at 16,000*g* for 20 min. The supernatants were then dried using a SpeedVac (Eppendorf, Vacufuge) and reconstituted in 400 μl of 80% methanol per sample by 10 min of water-bath sonication (Branson, 2800). Samples were centrifuged at 16,000*g* for 20 min, and 5 μl of the supernatant was used for LC–MS injection. Meanwhile, the remaining medium was removed, and cells were washed with 2 ml of cold PBS, then extracted with 1 ml of 80% methanol and placed on ice for 1 h. The extracts were centrifuged at 16,000*g* for 15 min, and 5 μl of the supernatant was used for LC–MS injection. LC was performed using an XBridge BEH Amide XP column (Waters, 186006724) under negative ion mode, as described above for mevalonate pathway metabolite measurements. MS was carried out using a Q Exactive Orbitrap mass spectrometer system (Thermo), and data were processed and analysed using TraceFinder software (Thermo, OPTON-31001).

### RNA-seq and data analysis

Total RNA was extracted from SORD WT (Cas9-expressing control cells) and KO cells derived from HCT116 and DLD1 using the RNeasy Plus Mini Kit (Qiagen, 74136). Before RNA extraction, cells were cultured under Glu + Fru conditions for 48 h. Sample quality control, RNA-seq and data analysis were performed by Novogene. In brief, after isolation, RNA integrity and quantification were assessed using the RNA Nano 6000 Assay Kit with the Bioanalyzer 2100 system (Agilent Technologies). Qualified samples had an RNA Integrity Number value of >10. Libraries were generated using the NEBNext Ultra RNA Library Prep Kit for Illumina (New England Biolabs, E7530L) following the manufacturer’s protocols, with index codes added. Clustering of the index-coded samples was performed on a cBot Cluster Generation System using the PE Cluster Kit cBot-HS (Illumina), according to the manufacturer’s instructions. After cluster generation, library preparations were sequenced on an Illumina platform to generate paired-end reads. Following quality control checks for Q20, Q30 and GC content, the paired-end clean reads were aligned to the reference genome using STAR (Spliced Transcripts Alignment to a Reference). Gene-level quantification was performed with FeatureCounts, and differential expression analysis was conducted using the DESeq2 R package. Differentially expressed genes were identified using the edgeR R package; those with a false discovery rate of <0.05 and |log_2_(fold change)| > 0.5 were used for pathway analysis with Qiagen Ingenuity Pathway Analysis. Heatmaps were generated using Qlucore (v.3.9). The R code used to process the RNA-seq data is available upon request.

### Analysis of data from public databases

Gene expression data from human CRCs and adjacent normal tissues (GSE41258, GSE14297, GSE49355 and GSE35834) were obtained from the Gene Expression Omnibus (https://www.ncbi.nlm.nih.gov/geo). Data from The Cancer Genome Atlas Pan-Cancer dataset were obtained from the UCSC Xena database. OncoGEO B37 and B38 datasets were obtained from QIAGEN OmicSoft Lands. The expression levels of SORD and AKR1B1 in normal tissue, primary tumour and metastatic tumour groups were extracted from these datasets and compared using unpaired *t*-tests or one-way ANOVA. Analysis of scRNA-seq data was performed using the Human Colon Cancer Atlas (c295) dataset via the Single Cell Portal (https://singlecell.broadinstitute.org/single_cell)

### Statistics and reproducibility

All animals were randomly grouped. No statistical methods were used to pre-determine sample sizes, but our sample sizes are similar to those reported in previous publications^9,33^. Data collection and analysis in mice were not conducted blind to the experimental conditions. In the animal experiments, mice that died prematurely or failed to develop primary colon tumours (owing to injection issues) were excluded from analysis. In Extended Data Fig. 3a,g (public gene expression data), one outlier data point (|x − μ| > 6σ) per figure was excluded for graphical purposes, and this exclusion did not affect the *P* value or interpretation. No data were excluded from other analyses.

All data are shown as means; error bars, s.e.m. Data distribution was assumed to be normal, but this was not formally tested. When comparing means between two groups, a two-tailed unpaired *t*-test was used after confirming that the data were sampled from a Gaussian distribution using the D’Agostino–Pearson normality test. When comparing means across more than two groups, a one-way ANOVA was performed using Prism 10 (GraphPad). For comparing the effects of genotype and treatment, two-way ANOVA was conducted with post hoc comparisons using Holm’s multiple comparisons test in Prism 10 (GraphPad). Statistical significance is shown as *P < 0.05, **P < 0.01, ***P < 0.001 and ****P < 0.0001. The Venn diagrams in Fig. 2 and Extended Data Fig. 3 were generated in https://bioinformatics.psb.ugent.be/webtools/Venn/.

### Reporting summary

Further information on research design is available in the Nature Portfolio Reporting Summary linked to this article.

## Supplementary information

Reporting Summary

## Source data

Source Data Fig. 1 Statistical source data.

Source Data Fig. 2 Statistical source data.

Source Data Fig. 3 Statistical source data.

Source Data Fig. 4 Statistical source data.

Source Data Extended Data Fig. 1 Statistical source data.

Source Data Extended Data Fig. 2 Statistical source data.

Source Data Extended Data Fig. 3 Statistical source data.

Source Data Extended Data Fig. 4 Statistical source data.

Source Data Extended Data Fig. 5 Statistical source data.

Source Data Extended Data Fig. 6 Statistical source data.

Source Data Extended Data Fig. 7 Statistical source data.

Source Data Extended Data Fig. 8 Statistical source data.

Source Data Extended Data Fig. 9 Statistical source data.

Source Data Extended Data Fig. 10 Unprocessed western blots and/or gels.

## Data Availability

The RNA-seq data have been deposited and are publicly available in the Sequence Read Archive (PRJNA1284926). Data supporting the findings of this study are available from the corresponding author upon reasonable request. Source data are provided with this paper.
