# Supplementary material for: Fructose and glucose from sugary drinks enhance colorectal cancer metastasis via SORD
Source: Nat Metab. 2025 Sep 19;7(10):2018–32. doi: 10.1038/s42255-025-01368-w (PMC12552132; doi:10.1038/s42255-025-01368-w)
Supplement: Supplementary file 1 — Reporting Summary [file 42255_2025_1368_MOESM1_ESM.pdf]

nature portfolio

Corresponding author(s): Jihye Yun

Last updated by author(s): 7/30/2025

Reporting Summary

Nature Portfolio wishes to improve the reproducibility of the work that we publish. This form provides structure for consistency and transparency in reporting. For further information on Nature Portfolio policies, see our Editorial Policies and the Editorial Policy Checklist.

Statistics

For all statistical analyses, confirm that the following items are present in the figure legend, table legend, main text, or Methods section.

n/a	Confirmed	
☐	☒	The exact sample size (*n*) for each experimental group/condition, given as a discrete number and unit of measurement
☐	☒	A statement on whether measurements were taken from distinct samples or whether the same sample was measured repeatedly
☐	☒	The statistical test(s) used AND whether they are one- or two-sided<br>*Only common tests should be described solely by name; describe more complex techniques in the Methods section.*
☒	☐	A description of all covariates tested
☐	☒	A description of any assumptions or corrections, such as tests of normality and adjustment for multiple comparisons
☐	☒	A full description of the statistical parameters including central tendency (e.g. means) or other basic estimates (e.g. regression coefficient) AND variation (e.g. standard deviation) or associated estimates of uncertainty (e.g. confidence intervals)
☐	☒	For null hypothesis testing, the test statistic (e.g. *F*, *t*, *r*) with confidence intervals, effect sizes, degrees of freedom and *P* value noted<br>*Give P values as exact values whenever suitable.*
☒	☐	For Bayesian analysis, information on the choice of priors and Markov chain Monte Carlo settings
☒	☐	For hierarchical and complex designs, identification of the appropriate level for tests and full reporting of outcomes
☒	☐	Estimates of effect sizes (e.g. Cohen's *d*, Pearson's *r*), indicating how they were calculated

Our web collection on statistics for biologists contains articles on many of the points above.

Software and code

Policy information about availability of computer code

Data collection	The IVIS data were collected using Spectral Instruments Imaging Aura Software (Version 4.0.7). The transwell, invasion and colony formation images were collected using Olympus cellSens Standard (Version 2.3). The NADH peredox reporter imaging data were collected using Nikon NIS Elements Imaging Software (Version 5.42.03). The SYBR green cell growth assay and enzymatic NAD+/NADH assay data were collected using Agilent BioTek Gen5 (Version 3.12). The metabolomics and other data with HPLC/MS were collected using Thermo Scientific Xcalibur (Version 4.2.47) and Thermo Scientific Freestyle (Version 1.5.93.34). The western blotting images were collected using Bio-Rad Image Lab Touch Software (Version 3.0.1.14). The Q-PCR data were collected using Bio-Rad CXF Manager (Version 3.1).
Data analysis	The statistical tests and graph were performed using GraphPad Prism (Version 10.1.2) and Microsoft Excel for Microsoft 365 MSO (Version 2308). The RNA-seq data and metabolomics data were analyzed with R (Version 4.2.1) and RStudio (Version 2023.12.0). The R package used including DESeq2 (Version 1.42.1), edgeR (Version 3.38.4) and dplyr (Version 1.0.10). The pathway analysis of RNA-seq data were analyzed with Qiagen Ingenuity Pathway Analysis (Version 111725566). The transwell and invasion assay data were analyzed with mageJ (Version 1.54f). The analysis of single cell RNA-seq database Human Colon Cancer Atlas (c295) was done with the Single Cell Portal (https://singlecell.broadinstitute.org/single_cell). The metabolomics and other data with HPLC/MS were analyzed using Thermo Scientific TraceFinder (Version 5.0.899.0). The Venn diagram of metabolomics data was generated with website https://bioinformatics.psb.ugent.be/webtools/Venn/. The IVIS data were analyzed using Aura Software (Version 4.0.7). The NADH peredox reporter imaging data were analyzed using Nikon NIS Elements Imaging Software (Version 5.42.03). The heatmap was drawn with Qlucore (Version 3.9).

For manuscripts utilizing custom algorithms or software that are central to the research but not yet described in published literature, software must be made available to editors and reviewers. We strongly encourage code deposition in a community repository (e.g. GitHub). See the Nature Portfolio guidelines for submitting code & software for further information.

Data

Policy information about availability of data

All manuscripts must include a data availability statement. This statement should provide the following information, where applicable:

- Accession codes, unique identifiers, or web links for publicly available datasets
- A description of any restrictions on data availability
- For clinical datasets or third party data, please ensure that the statement adheres to our policy

The SORD and AKR1B1 mRNA expression data shown in Fig. 3a and Extended Data Fig. 3 were obtained from publicly available datasets. Specifically, data were retrieved from the TCGA Pan-Cancer database (https://xenabrowser.net/datapages/?cohort=TCGA%20Pan-Cancer%20(PANCAN)&removeHub=https%3A%2F%2Fxena.treehouse.gi.ucsc.edu%3A443), and from the Gene Expression Omnibus under accession codes GSE41258, GSE14297, GSE49355, and GSE35834.

Additional datasets were accessed via the QIAGEN OmicSoft Lands platform under accession codes OncoGEO_B37 and OncoGEO_B38.The single-cell RNA sequencing data from the Human Colon Cancer Atlas (c295), also shown in Extended Data Fig. 3, are publicly available on the Single Cell Portal (https://singlecell.broadinstitute.org/single_cell).

The RNA-seq datasets generated and analyzed during this study are available in the Sequence Read Archive under accession number PRJNA1284926.

Research involving human participants, their data, or biological material

Policy information about studies with human participants or human data. See also policy information about sex, gender (identity/presentation), and sexual orientation and race, ethnicity and racism.

Reporting on sex and gender	Sex and gender were not provided or used in the selection of slides for histological analysis.
Reporting on race, ethnicity, or other socially relevant groupings	No race, ethnicity, or other socially relevant characteristics were considered in the analysis.
Population characteristics	Human histology slides were obtained from Human Tissue Acquisition & Pathology (HTAP) Services in Baylor College Medicine. All the samples were from patients diagnosed with colorectal cancer at the Michael E. DeBakey Veteran's Affairs Hospital, Ben Taub General Hospital, and Baylor St. Luke's Medical Center and grouped based on the grades of cancer. No other population characteristics were considered in the analysis.
Recruitment	All the samples were collected with patient consent. The participants were recruited only based on the stage of colorectal cancer. No bias will be present to impact the results.
Ethics oversight	The collection of patient-derived samples was approved by Baylor College Medicine.

Note that full information on the approval of the study protocol must also be provided in the manuscript.

Field-specific reporting

Please select the one below that is the best fit for your research. If you are not sure, read the appropriate sections before making your selection.

☒ Life sciences ☐ Behavioural & social sciences ☐ Ecological, evolutionary & environmental sciences

For a reference copy of the document with all sections, see nature.com/documents/nr-reporting-summary-flat.pdf

Life sciences study design

All studies must disclose on these points even when the disclosure is negative.

Sample size	No statistical methods were used to predetermine sample size. The sample sizes were determined based on standard experimental group sizes to achieve acceptable power. In general, 3-6 biological replicates are used for in vitro experiments with cultured cells and 8-15 biological replicates for in vivo experiments with mouse models. In the in vivo experiments, sample sizes might be different in different groups because of limited the success rate of mice surgery and the unavoidable cancer related animal death.<br>Reference: Naegle, Kristen, Nancy R. Gough, and Michael B. Yaffe. "Criteria for biological reproducibility: what does "n" mean?." Science signaling 8.371 (2015): fs7-fs7.
Data exclusions	In the animal experiments, data from the mice which died before the sacrificing date or without tumors (CRC animal models failed) were excluded.<br>In extended Data Fig. 3a and 3g, one outlier data point ($\lvert x-\mu \rvert > 6\sigma$) per figure was excluded (the exclusion doesn't affect the p value and significance). No data were excluded from other analyses.
Replication	All experiments were performed independently at least two times with the exception of the RNA-seq and certain animal experiments.

Randomization	Samples were randomly assigned to groups.
Blinding	Blinding was not performed because it was difficult to mask the experimenters from knowing which cages received specific treatments due to clear labeling and color coding. However, all pathological evaluations were conducted in a blinded manner.

Reporting for specific materials, systems and methods

We require information from authors about some types of materials, experimental systems and methods used in many studies. Here, indicate whether each material, system or method listed is relevant to your study. If you are not sure if a list item applies to your research, read the appropriate section before selecting a response.

Materials & experimental systems

n/a	Involved in the study
☐	☒ Antibodies
☐	☒ Eukaryotic cell lines
☒	☐ Palaeontology and archaeology
☐	☒ Animals and other organisms
☒	☐ Clinical data
☒	☐ Dual use research of concern
☒	☐ Plants

Methods

n/a	Involved in the study
☒	☐ ChIP-seq
☒	☐ Flow cytometry
☒	☐ MRI-based neuroimaging

Antibodies

Antibodies used	Primary antibody for the immunohistochemistry staining: SORD (Sigma, HPA040621).<br>Primary antibodies for the western blotting: SORD (1:1000, Proteintech, 15881-1-AP), AKR1B1 (1:1000, Proteintech, 15439-1-AP) and β-actin (1:2000, Cell Signaling Technology, 3700).<br>Secondary antibodies: Goat anti-Rabbit IgG(H+L)-HRP (1:5000, GenDepot, SA002) and Goat anti-Mouse IgG(H+L)-HRP (1:5000, GenDepot, SA001)
Validation	SORD (Sigma, HPA040621) was validated by Sigma using human liver, kidney, prostate and skeletal muscle immunohistochemistry staining.<br>SORD (Proteintech, 15881-1-AP) was validated by Proteintech using HeLa cell, mouse liver and rat liver western blotting.<br>AR (Proteintech, 15439-1-AP) was validated by Proteintech using western blotting of sh-Control and sh-AKR1B1 transfected A431 cells.<br>β-actin (Cell Signaling Technology, 3700) was validated by Proteintech using western blotting of COS, HeLa, C2C12, C6 and CHO cell lines.

Eukaryotic cell lines

Policy information about cell lines and Sex and Gender in Research

Cell line source(s)	The following colorectal cancer (CRC) cell lines were sourced from the American Type Culture Collection (ATCC): HCT116 (ATCC, CCL-247), DLD1 (ATCC, CCL-221), RKO (ATCC, CRL-2577), HCT8 (ATCC, CCL-244), NCI-H508 (ATCC, CCL-253), Colo205 (ATCC, CCL-222), SW620 (ATCC, CCL-227), SW48 (ATCC, CCL-231), and HCT15 (ATCC, CCL-225). The DiFi, GP5D, and HT55 cell lines were provided by Dr. Jeffrey Engelman, and the VACO432 cell line was provided by Dr. Sandy Markowitz.<br>All the gene modified cell lines were constructed as described in the Methods part.
Authentication	All cell lines were authenticated with STR genetic testing.
Mycoplasma contamination	Cell lines routinely tested negative for mycoplasma by the mycoplasma detection kit (Lonza Walkersville, LT07-710).
Commonly misidentified lines<br>(See ICLAC register)	No cell lines used in this study were found in the database of commonly misidentified cell lines that is maintained by ICLAC and NCBI Biosample.

Animals and other research organisms

Policy information about studies involving animals; ARRIVE guidelines recommended for reporting animal research, and Sex and Gender in Research

Laboratory animals	7-week-old male NU/J mice were purchased from the Jackson Laboratory (the Jackson Laboratory, Strain #: 002019).<br>Male and female NOD.Cg-Prkdcscid Il2rgtm1Wjl/SzJ (NSG) mice were purchased from the Jackson Laboratory (the Jackson Laboratory, Strain #: 005557).
Wild animals	No wild animal was included in this study.

Reporting on sex	All in vivo experiments were conducted using male mice, as specified in the manuscript. Both male and female NSG mice were used for breeding purposes only. The primary focus of this study is on in vitro cellular mechanisms; in vivo xenograft models were used to assess cell-autonomous effects. We do not anticipate sex-based differences in the outcomes relevant to this study.
Field-collected samples	No Field-collected sample was included in this study.
Ethics oversight	All animal studies were approved by the Institutional Animal Care and Use Committee (IACUC) of Baylor College of Medicine and MD Anderson Cancer Center.

Note that full information on the approval of the study protocol must also be provided in the manuscript.

Plants

Seed stocks	N/A
Novel plant genotypes	N/A
Authentication	N/A
