# Supplementary figures and images for: Fructose and glucose from sugary drinks enhance colorectal cancer metastasis via SORD
Source: Nat Metab. 2025 Sep 19;7(10):2018–32. doi: 10.1038/s42255-025-01368-w (PMC12552132; doi:10.1038/s42255-025-01368-w)

**β-Actin**

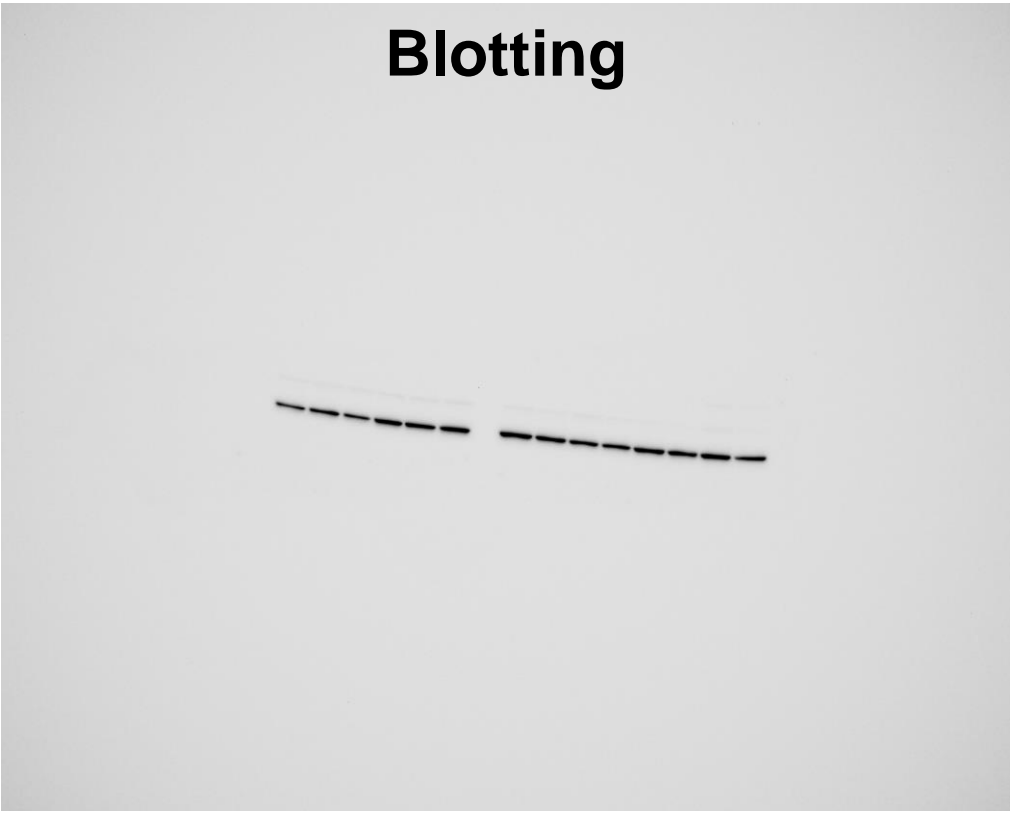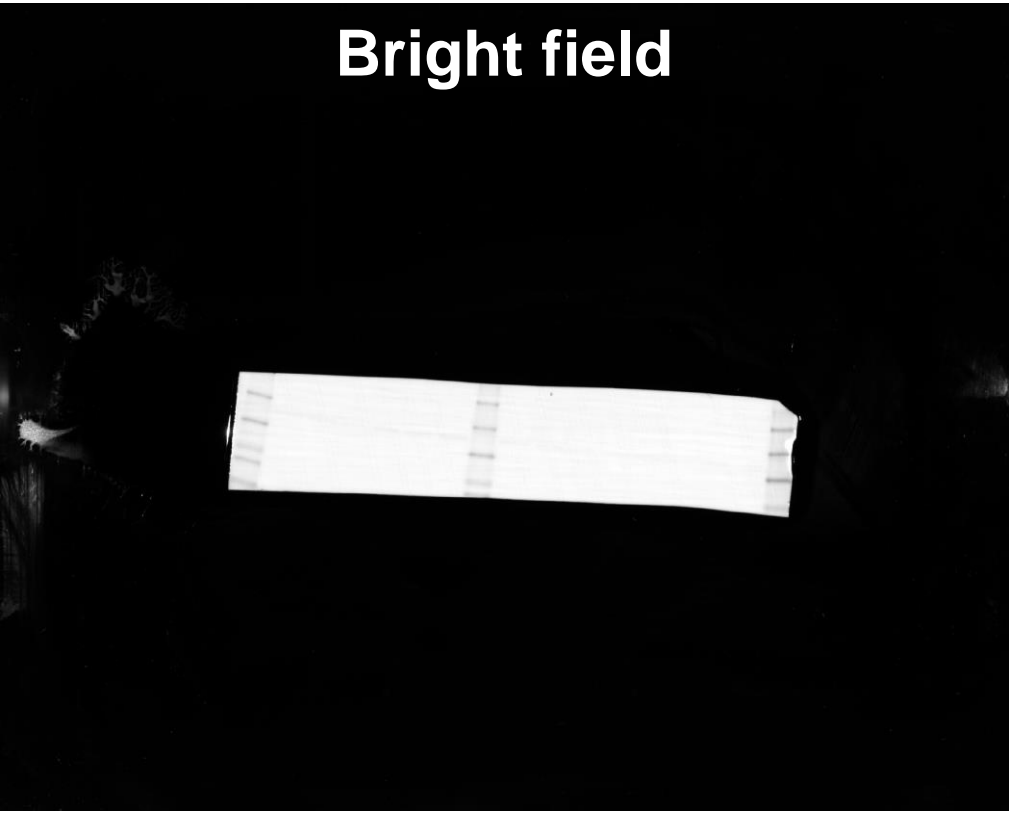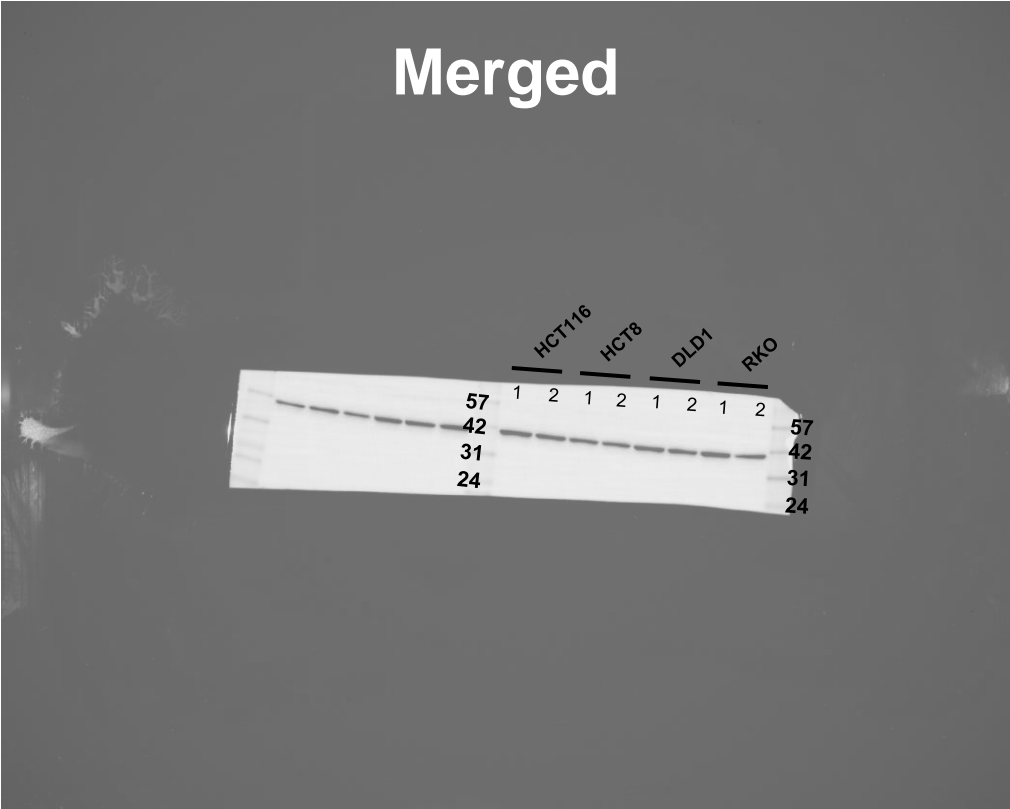

**SORD**

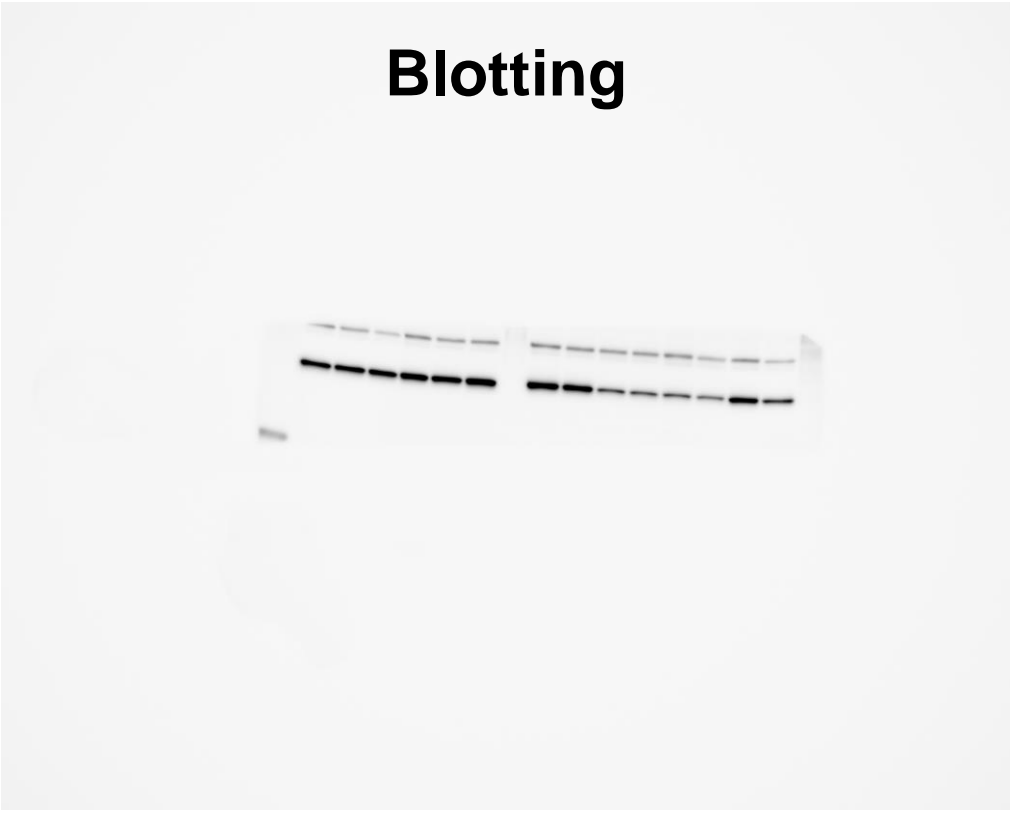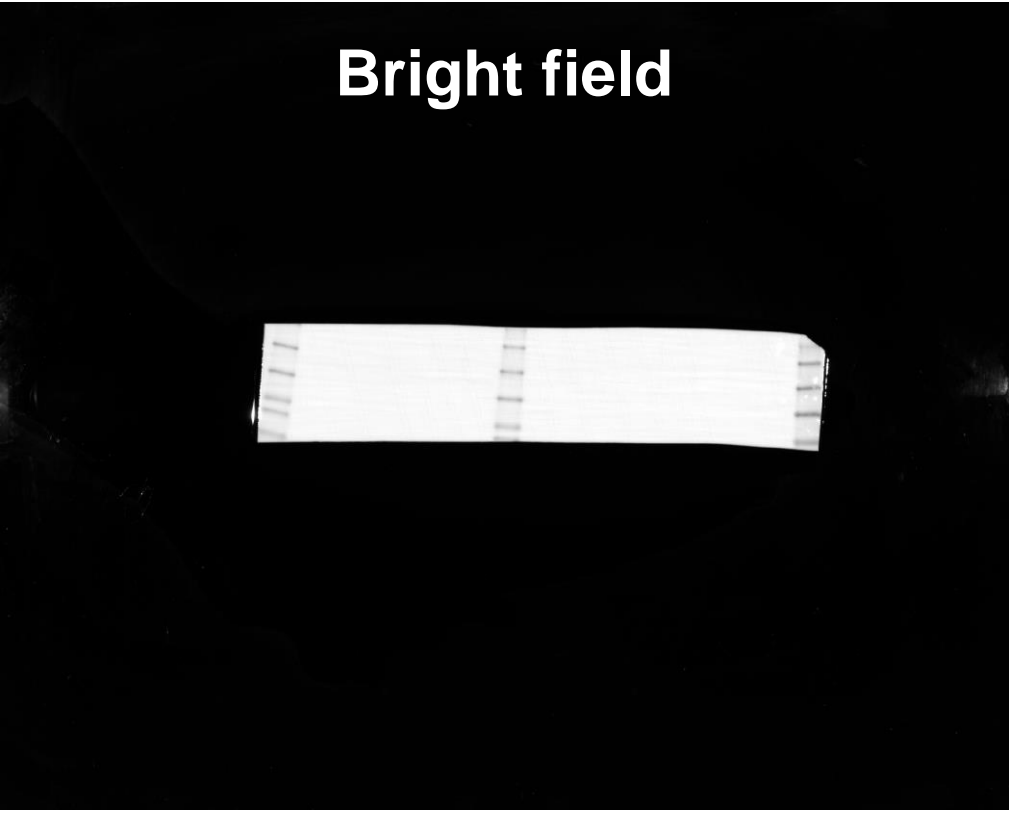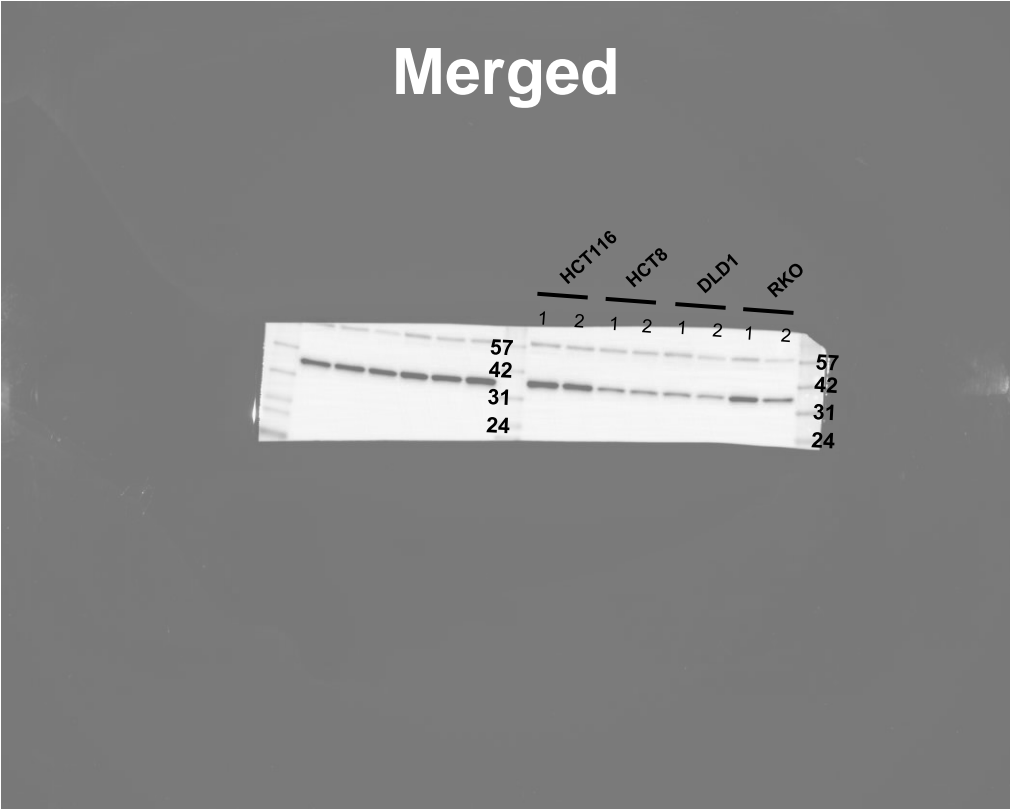

Blotting

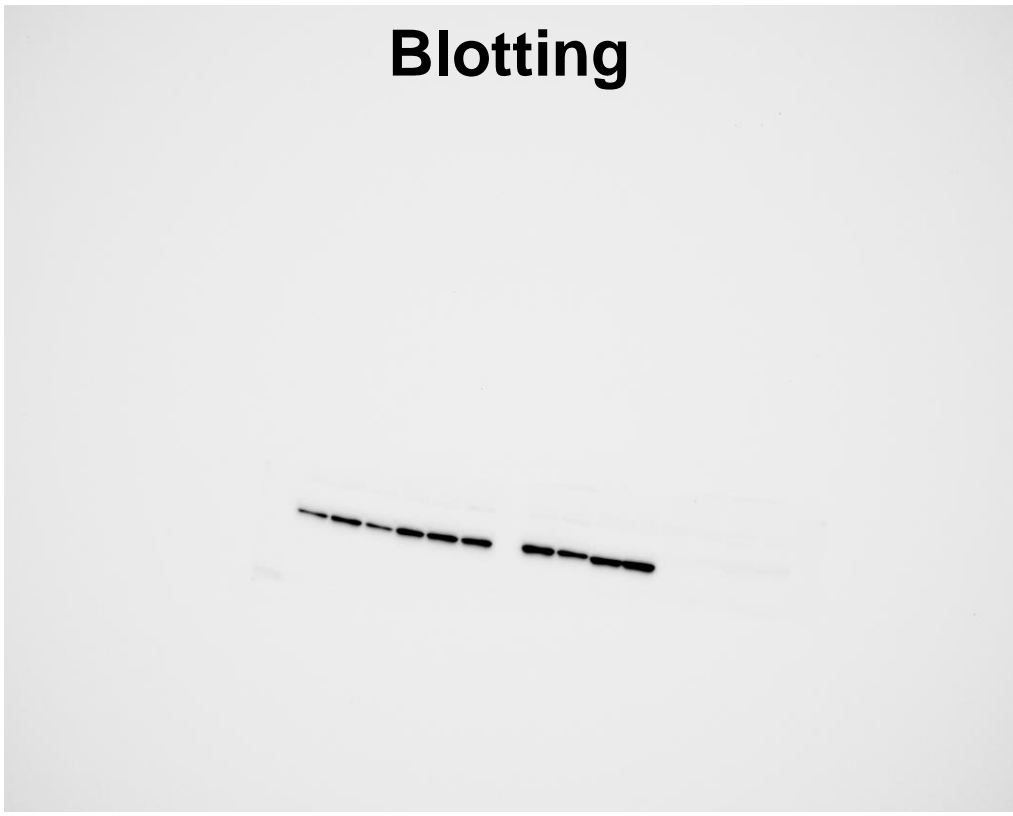

Bright field

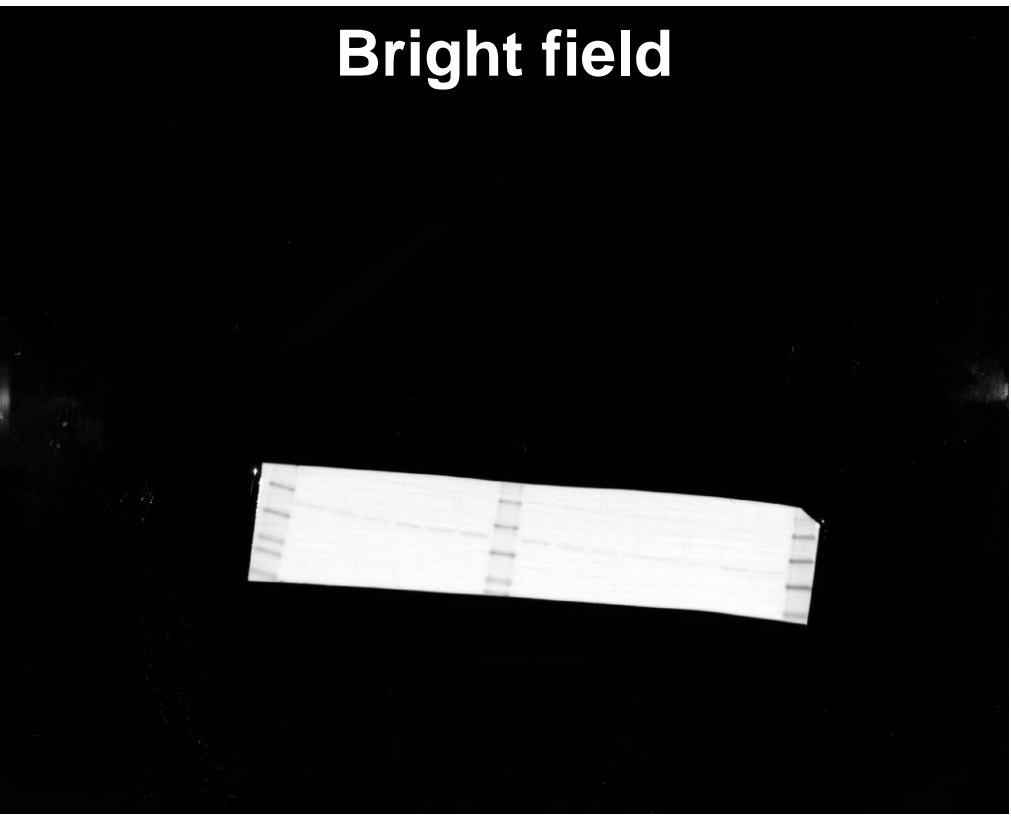

Merged

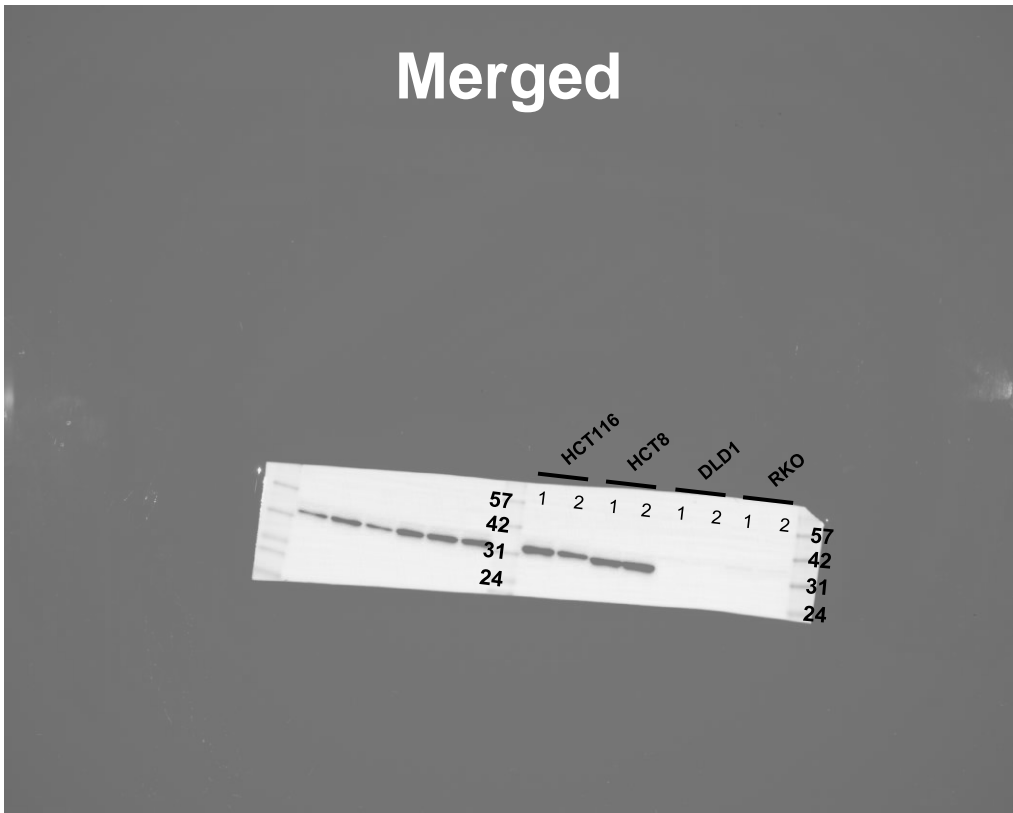

AR

Supplement: Supplementary file 15 — Unprocessed western blots and/or gels. [file 42255_2025_1368_MOESM15_ESM.pdf]
